# Diagnosis, management, and prevention of bronchiolitis in primary care: a survey of Italian family paediatricians

**DOI:** 10.1186/s13052-025-02152-y

**Published:** 2025-11-19

**Authors:** Marina Picca, Chiara Pezzini, Elena Baggi, Paola Manzoni, Antonella Mezzopane, Adriano La Vecchia, Gregorio Paolo Milani

**Affiliations:** 1 Italian Primary Care Paediatrics Society (SiCuPP), Lombardy, Italy; 2 Pediatric Unit, Fondazione IRCCS Ca’ Granda Ospedale Maggiore Policlinico, Via della Commenda, 9, 20122 Milan, MI Italy; 3 Department of Medicine and Surgery, University of Milan-Bicocca, Monza, Italy; 4 Department of Clinical Sciences and Community Health, Università Degli Studi Di Milano, 20122 Milan, Italy

## Abstract

**Background:**

Primary care paediatricians play a key role in the diagnosis, management, and prevention of bronchiolitis, but data on their clinical practices remain limited.

**Methods:**

An online survey was conducted via email by the Lombardy section of the Italian Primary Care Paediatrics Society (SICuPP) between January 1 and April 30, 2025. Primary care paediatricians were invited to participate via email. Associations were assessed using the chi-square or Fisher’s exact test. Multivariable logistic regression identified factors associated with the prescription of antibiotics, corticosteroids, and bronchodilators—three drug categories commonly used in bronchiolitis despite not being recommended by current guidelines.

**Results:**

The response rate was 28.8%, yielding 306 valid responses. Most respondents (62.1%) had over 20 years of clinical experience. Diagnostic criteria varied: 42.8% used 12 months as the upper age limit, 42.2% used 24 months, and 5.6% applied no age limit. Diagnostic approaches differed, with 45.1% relying on wheezing/grunting and 36.9% on wet sounds. Pulse oximetry was always used by 39.5%, and 67.9% never used rapid viral tests. Bronchodilators and steroids were recommended by 37.6% and 32.3%, respectively. Antibiotics were prescribed in 30.1% of cases with poor general condition and 18.6% with fever. Nirsevimab prophylaxis was well accepted (96.4%), with high caregiver compliance (97.7%). Paediatricians diagnosing bronchiolitis up to 36 months were less likely to report never prescribing antibiotics (OR 0.13, 95% CI 0.02–0.51), whereas those relying on widespread sounds were more likely than those using grunting or wheezing (OR 2.42, 95% CI 1.23–4.83). Bronchodilator use was lower with diagnosis based on widespread sounds (OR 0.38, 95% CI 0.18–0.77). Steroid use was higher without an age limit (OR 3.09, 95% CI 1.09–9.25) and lower with widespread sounds (OR 0.31, 95% CI 0.13–0.66).

**Conclusions:**

This first Italian survey on bronchiolitis management in primary care reveals substantial variability in diagnostic and treatment practices. Diagnostic inconsistency is associated with non-guideline-recommended prescribing. Standardized diagnostic criteria are needed. Nirsevimab prophylaxis was widely accepted, supporting its continued use to reduce the burden of bronchiolitis.

**Supplementary Information:**

The online version contains supplementary material available at 10.1186/s13052-025-02152-y.

## Background

Bronchiolitis is an acute viral lower respiratory tract infection affecting infants and young children, with human respiratory syncytial virus (RSV) identified as the primary etiological agent [1]. Diagnosis is made clinically, based on characteristic signs and symptoms such as rhinorrhea, cough, respiratory distress, wheezing, and crackles [2, 3].

It constitutes a substantial global health burden in this age group, significantly contributing to both morbidity and mortality [4, 5]. In high-income countries, bronchiolitis is a leading cause of hospitalization and emergency department (ED) visits during the first years of life, accounting for up to 17% of hospital admissions and 15% of emergency presentations among infants in the United States [6]. The recent introduction of nirsevimab, a long-acting monoclonal antibody approved by the European Medicines Agency (EMA) in November 2022 [7, 8], is altering the epidemiology and severity of RSV-related bronchiolitis across Europe [9, 10].

A major challenge in the management of bronchiolitis is the adoption of evidence-based practices [1]. Nutritional measures, oxygen supplementation, and, when necessary, non-invasive ventilation remain the only evidence-based therapeutic interventions for this condition [11–13]. A number of studies have highlighted a considerable heterogeneity in the management of infants with bronchiolitis both in EDs and in regular wards [14–16]. Despite the high prevalence of mild cases in infants, typically managed by primary care physicians, there remains a notable scarcity of research focused on bronchiolitis management within European primary care settings. As the first point of contact for most patients, primary care plays a critical role in enabling early intervention and potentially reducing disease severity and hospital utilization.

The present survey aims to address the existing knowledge gap by investigating how primary care paediatricians approach the diagnosis, treatment, and prevention of bronchiolitis. We hypothesize that significant heterogeneity exists in the management of bronchiolitis in this setting.

## Methods

### Data source and study design

We conducted an online survey via email, developed by the Lombardy section of the Italian Primary Care Paediatrics Society (Società Italiana delle Cure Primarie Pediatriche, SICuPP) [17], from January 1 to April 30, 2025. Following the initial email invitation, two additional reminders were sent after 2 and 4 weeks.

Lombardy, the most populous region in Italy, with over 1.5 million residents under the age of 18 [18], had 1,072 primary care paediatricians as of January 1, 2024, representing approximately one-sixth of the national total [19].

### Data collection and questionnaire

We developed a questionnaire that investigated four main topics: work experience, diagnostic approach, treatment, and prevention of bronchiolitis. Each question allowed only a single-choice response. The questionnaire was developed after a literature review and then pre-tested by five paediatricians to evaluate the clarity of the questions (overall agreement > 95%). Then, a test-retest was performed, including four additional paediatricians to assess the reproducibility. They answered the survey twice after a 14-day interval. The intra-rater reproducibility was > 96%. A full copy of the final version of the survey and its English translation is provided in *Appendix A* (Supplementary Materials 1).

### Statistical analysis

A preliminary descriptive analysis was conducted, with categorical variables summarized using absolute and relative frequencies. Associations between categorical variables were assessed using the chi-square test or Fisher’s exact test, as appropriate. Multivariable logistic regression models were employed to identify factors associated with the prescription of antibiotics, corticosteroids, and bronchodilators. These three non-guideline-recommended drug categories were chosen since previous studies conducted in ED and regular wards showed they are frequently employed in these settings [14, 15, 20]. Candidate predictors were prespecified a priori based on the hypothesis that heterogeneity in diagnostic criteria could influence treatment decisions [21]. For each multivariable logistic regression model, multicollinearity was assessed using variance inflation factors (VIF), goodness-of-fit using the Hosmer–Lemeshow test, and calibration using the calibration intercept and slope obtained from regressions of observed outcomes on the logit of predicted probabilities. Robust standard errors were not applied, as the unit of analysis was the physician. Clustering at the physician level was not applicable, as the unit of analysis was the individual physician; robust standard errors were therefore not applied. Statistical significance was defined as a p-value < 0.05. All analyses were conducted using R software (version 4.3.2 for Windows).

### Ethical consideration

This study was conducted in accordance with the latest version of the Declaration of Helsinki (October 2024) [22]. Informed consent was obtained from all participants, and no personally identifiable information was collected, thereby ensuring participant anonymity. According to current Italian legislation, observational studies based on anonymous survey data and not involving patients directly do not require approval from an ethics committee (Article 110, Italian Data Protection Code, as amended by the ‘PNRR bis’ decree). The study adheres to the STROBE reporting guidelines for observational studies.

## Results

We collected 309 responses, representing a response rate of 28.8% from the 1,072 primary care paediatricians practicing in Lombardy. Three respondents were excluded for not providing consent, resulting in a final sample of 306 participants. Among them, 42 (13.7%) had less than 10 years of clinical experience, 74 (24.2%) had between 10 and 20 years, and 190 (62.1%) had more than 20 years of experience. Table 1 summarizes the frequencies and percentages of answers regarding paediatricians’ experiences and bronchiolitis diagnosis. Item-level missingness was minimal, and denominators are reported in the tables.

**Table 1:** Frequency and percentage of respondents for each survey item on pediatrician experience and bronchiolitis diagnosis (*n* = 306)

Survey Items on Pediatric Experience and Diagnosis with Response Options	*N* (%)
**How many years have you been practicing as a family pediatrician?**	**306 (100)**
Less than 10 years	42 (13.7)
10–20 years	74 (24.2)
More than 20 years	190 (62.1)
**DIAGNOSIS**	
**What is the upper age limit you consider for diagnosing bronchiolitis?**	**306 (100)**
≤6 months	16 (5.2)
≤12 months	131 (42.8)
≤24 months	129 (42.2)
≤36 months	13 (4.2)
I do not consider any upper age limit	17 (5.6)
**Given the age cut-off you consider appropriate**,** in which cases do you diagnose bronchiolitis?**	**306 (100)**
Widespread sounds on auscultation of the lower airways, regardless of their type	55 (18.0)
Signs of respiratory distress with grunting and/or wheezing on auscultation of the lower airways	138 (45.1)
Signs of respiratory distress with widespread wet sounds on auscultation of the lower airways (crackles or rales)	113 (36.9)
**Given the selected criteria**,** during which periods of the year do you consider a diagnosis of bronchiolitis?**	**306 (100)**
Only during the epidemic period of respiratory viruses typically associated with bronchiolitis (autumn–winter)	64 (20.9)
Only during the epidemic period of respiratory viruses typically associated with bronchiolitis and in spring	122 (39.9)
Throughout the entire year	120 (39.2)
**Do you measure the respiratory rate in patients diagnosed with bronchiolitis?**	**306 (100)**
Never	6 (2.0)
Only in cases of severe respiratory distress	105 (34.3)
Always	195 (63.7)
**Do you use pulse oximetry to monitor patients diagnosed with bronchiolitis?**	**306 (100)**
Never	17 (5.6)
In selected cases based on the availability of a pulse oximeter probe suitable for the child’s finger	119 (38.9)
Only in cases of severe respiratory distress	49 (16)
Always	121 (39.5)
**Do you perform rapid tests for the etiological diagnosis of bronchiolitis?**	**305 (99.7)**
Never	207 (67.9)
Occasionally	60 (19.7)
Always	38 (12.5)
**Which tests do you perform?**	**98 (32)**
For the detection of RSV	6 (6.1)
For the detection of RSV and influenza	9 (9.2)
For the detection of RSV, influenza, and SARS-CoV-2	18 (18.4)
For the detection of RSV, influenza, SARS-CoV-2, and other viruses/bacteria	65 (66.3)

Denominators vary for individual questions due to item-level nonresponse. Some questions were only applicable to participants who answered specific preceding questions (skip patterns). All denominators are reported in the table

Abbreviation: RSV, Respiratory Syncytial Virus, SARS-CoV-2, Severe acute respiratory syndrome coronavirus 2

### Diagnosis

Age thresholds for diagnosing bronchiolitis varied: 16 (5.2%) used < 6 months, 131 (42.8%) < 12 months, 129 (42.2%) < 24 months, and 17 (5.6%) reported no limit. Diagnostic criteria were also heterogeneous: 55 (18.0%) relied on widespread adventitious sounds, 138 (45.1%) on respiratory distress with grunting/wheezing, and 113 (36.9%) on distress with moist sounds. Bronchiolitis was considered seasonal by 186 (60.8%) respondents, while 120 (39.2%) considered it year-round. Respiratory rate was always measured by 195 (63.7%), and pulse oximetry by 121 (39.5%); 208 (67.9%) never used rapid viral tests.

### Management and referral

In outpatient care, almost all paediatricians recommended monitoring general condition 286 (93.5%) and feeding 293 (95.7%). Hypertonic saline aerosols were used by 198 (64.7%), bronchodilators by 115 (37.6%), and steroids by 99 (32.3%). Referral to the ED was most frequently recommended for dehydration or inadequate feeding 270 (88.2%), low family compliance 266 (86.9%), and severe distress regardless of oxygen level 258 (84.3%). Referral criteria based on oxygen saturation varied: 191 (62.4%) used a 92% threshold, while 79 (25.8%) used < 90%.

Antibiotics were never prescribed by 158 (51.6%), but were used in selected situations: poor general condition 92 (30.1%), fever 57 (18.6%), severe distress 44 (14.4%), age < 3 months 39 (12.7%), age < 1 month 34 (11.1%), and reduced feeding 7 (2.3%).

Main reported difficulties were avoiding unnecessary drug prescriptions 182 (59.5%), managing patients in outpatient settings 94 (30.7%), and diagnostic differences between ambulatory and hospital care 143 (46.7%). Problems with pulse oximeter probe size were noted by 144 (47.1%). Table 2 presents the frequencies and percentages of responses on management, referral, and treatment.

**Table 2:** Frequency and percentage of responses for each survey item on Management, Referral, and treatment (*n* = 306)

Survey Items on Management, Referral, and Treatment with Response Options	*N* (%)
**In the outpatient management of bronchiolitis**,** what routine advice do you give to parents?**	**306 (100)**
Monitoring of general condition/well-being	286 (93.5)
Monitoring of nutritional intake/hydration	293 (95.7)
Use of bronchodilators	115 (37.6)
Use of inhaled or oral steroids	99 (32.3)
Use of hypertonic saline aerosol	198 (64.7)
Nasal irrigation	256 (83.7)
Other	11 (3.6)
**Under which of the following circumstances do you decide to refer a patient with bronchiolitis to the hospital?**	**306 (100)**
Oxygen saturation in room air < 90%	79 (25.8)
Oxygen saturation in room air < 92%	191 (62.4)
Episodes of apnea reported by the parent and/or observed during the visit	195 (63.7)
Significant respiratory distress regardless of a normal oxygen saturation reading	258 (84.3)
Dehydration and/or nutritional intake < 50% in the previous 24 h	270 (88.2)
Age < 1 month	181 (59.1)
Age < 3 months	124 (40.5)
Prematurity (< 35 weeks of gestational age) / chronic condition predisposing to severe bronchiolitis	231 (75.5)
Poor adherence or ability of the family to follow medical advice	266 (86.9)
Fever > 39 °C	88 (28.8)
Fever that does not respond to antipyretics	105 (34.3)
**Treatment recommendations**	
**Do you recommend the use of respiratory physiotherapy in patients with bronchiolitis?**	**306 (100)**
Never	196 (64)
Occasionally	98 (32)
Always	12 (3.9)
**Do you recommend the use of osteopathic treatments in patients with bronchiolitis?**	**306 (100)**
Never	288 (94.1)
Occasionally	17 (5.6)
Always	1 (0.3)
In which of the following cases do you usually prescribe prophylactic^*^ **antibiotics in patients with bronchiolitis?**	**306 (100)**
Always	2 (0.6)
In patients younger than 1 month of age	34 (11.1)
In patients younger than 3 months of age	39 (12.7)
In case of fever	57 (18.6)
In case of poor general condition	92 (30.1)
In case of reduced nutritional intake	7 (2.3)
In case of severe respiratory distress	44 (14.4)
Never	158 (51.6)
**What do you consider to be the greatest challenge in managing bronchiolitis in accordance with clinical guidelines?**	**306 (100)**
Difficulty in diagnosis	21 (6.9)
Difficulty refraining from prescribing medications	182 (59.5)
Difficulty in following up with the patient in the community	94 (30.7)
Different diagnostic and therapeutic approaches between community and hospital settings	143 (46.7)
Difficulty using the pulse oximeter probe, often not suitable for the finger of very small infants	144 (47.1)
Other	13 (4.2)

* The questions refer to antibiotic prescribing in bronchiolitis. The term “prophylactic” appears in the table only as in the original survey wording.

### Nirsevimab prophylaxis

Most paediatricians were in favor of nirsevimab for all infants 295 (96.4%). A total of 217 (70.9%) had participated in the most recent campaign, of whom 212 (97.7%) reported high caregiver compliance. Only 6 (2.8%) rated the experience as negative or very negative.

Looking ahead, 130 of the 217 paediatricians who participated in the last campaign (59.9%) were willing to take part again, and 84 (38.7%) would do so only if organizational aspects improved. Among the 89 non-participants, 11 (12.4%) expressed willingness to join in the future, while 40 (44.9%) would participate only with better organization. Table 3 presents the frequencies and percentages of responses on nirsevimab prophylaxis.

**Table 3:** Frequency and percentage of respondents for each survey item on nirsevimab prophylaxis (*n* = 306)

Survey Items on Nirsevimab Prophylaxis with Response Options	*N* (%)
**What do you think about the new prophylaxis with Nirsevimab for RSV infection?**	**306 (100)**
Favorable in all newborns/infants	295 (96.4)
Favorable only in selected cases	8 (2.6)
Not favorable	3 (1.0)
**The Nirsevimab prophylaxis program has been implemented in the clinic**	217 (70.9)
**How have most parents/caregivers responded to immunoprophylaxis with Nirsevimab?**	**217 (70.9)**
They immediately agreed to undergo the prophylaxis	212 (97.7)
They initially refused/opposed the prophylaxis but later accepted	5 (2.3)
**How would you describe your experience with immunoprophylaxis?**	**217 (70.9)**
Very negative	3 (1.4)
Negative	3 (1.4)
Positive	60 (27.6)
Very positive	151 (69.6)
**Would you be willing to repeat it in the upcoming season?**	**217 (70.9)**
I don’t know	3 (1.4)
Yes, but only under better organizational conditions	84 (38.7)
Yes	130 (59.9)
**Would you be willing to carry it out in the upcoming season?**	**89 (29.1)**
No	24 (27)
I don’t know	14 (15.7)
Yes, but only under better organizational conditions	40 (44.9)
Yes	11 (12.4)

Denominators vary for individual questions due to item-level nonresponse. Some questions were only applicable to participants who answered specific preceding questions (skip patterns). All denominators are reported in the table

Abbreviation: RSV, Respiratory Syncytial Virus

### Factors associated with non-guideline-recommended therapy prescriptions

Table 4 presents the factors associated with prescriptions of antibiotics, bronchodilators, and steroids.

**Table 4:** Multivariate logistic regression of guideline-non-recommended prescriptions

Dependent variable	Independent variables	OR	95%CI	*p*-value
Never prescribe antibiotics	≤ 6 months	0.71	0.24–2.05	0.5
	≤ 12 months	Reference		
	≤ 24 months	1.07	0.65–1.76	0.8
	≤ 36 months	0.13	0.02–0.51	0.01
	No upper age limit considered	0.65	0.22–1.81	0.4
	URI prodrome + respiratory distress with grunting and/or wheezing on lower lung auscultation	Reference		
	URI prodrome + diffuse lung sounds on lower auscultation (any type)	2.42	1.23–4.83	0.01
	URI prodrome + respiratory distress with diffuse wet sounds (e.g., crackles or rales) on lower auscultation	1.43	0.86–2.39	0.2
Prescription of bronchodilators	≤ 6 months	2.21	0.76–6.63	0.1
	≤ 12 months	Reference		
	≤ 24 months	1.02	0.61–1.71	0.9
	≤ 36 months	1.34	0.38–4.42	0.6
	No upper age limit considered	1.49	0.52–4.21	0.4
	URI prodrome + respiratory distress with grunting and/or wheezing on lower lung auscultation	Reference		
	URI prodrome + diffuse lung sounds on lower auscultation (any type)	0.38	0.18–0.77	0.009
	URI prodrome + respiratory distress with diffuse wet sounds (e.g., crackles or rales) on lower auscultation	0.69	0.41–1.15	0.15
Prescription of steroids	≤ 6 months	0.96	0.28–2.89	0.9
	≤ 12 months	Reference		
	≤ 24 months	0.98	0.57–1.69	0.9
	≤ 36 months	2.51	0.73–8.48	0.1
	No upper age limit considered	3.09	1.09–9.25	0.036
	URI prodrome + respiratory distress with grunting and/or wheezing on lower lung auscultation	Reference		
	URI prodrome + diffuse lung sounds on lower auscultation (any type)	0.31	0.13–0.66	0.003
	URI prodrome + respiratory distress with diffuse wet sounds (e.g., crackles or rales) on lower auscultation	0.61	0.36–1.05	0.074

Abbreviation: URI, Upper Respiratory Tract Infection

Paediatricians who diagnosed bronchiolitis up to 36 months were less likely to report that they never prescribe antibiotics (OR 0.13, 95% CI 0.02–0.51) compared to those using 12 months as the upper age limit. Conversely, those who based the diagnosis on widespread sounds, regardless of type, were more likely to report that they never prescribe antibiotics (OR 2.42, 95% CI 1.23–4.83) compared to those who considered grunting and/or wheezing. The prescription of bronchodilators was less common among those who diagnosed bronchiolitis based on widespread sounds regardless of type, compared to those who considered grunting and/or wheezing (OR 0.38, 95% CI 0.18–0.77). Steroid use was more common among those who did not use an age limit for diagnosis compared to those who used 12 months (OR 3.09, 95% CI 1.09–9.25), and less common among those using widespread sounds as diagnostic criteria compared to those using grunting and/or wheezing (OR 0.31, 95% CI 0.13–0.66). R log outputs from the logistic regression models are provided in Supplementary Materials 2. All three models showed adequate fit (Hosmer–Lemeshow *p* > 0.05), no multicollinearity concerns (Max VIF ≤ 1.01), and good calibration (intercept near 0, slope near 1). Detailed diagnostic assessments are reported in *Appendix B* (Supplementary Materials 1).

## Discussion

To our knowledge, this is the first survey specifically targeting primary care paediatricians in Italy that investigates bronchiolitis from diagnosis to management and prevention. The main findings of this survey can be summarized as follows: (1) There is a considerable heterogeneity in the criteria applied for the diagnosis of bronchiolitis in infants among primary care paediatricians, (2) This heterogeneity is highly associated with different, sometimes not evidence-based, therapeutic approaches, (3) The experience of primary care paediatricians in the administration of nirsevimab prophylaxis was overall positive.

The results highlight a high degree of heterogeneity in the diagnostic criteria used. More than 40% of respondents reported using either 12 or 24 months as the upper age limit for diagnosing bronchiolitis, while 36.9% considered “wet sounds” and 45.1% included grunting or wheezing as diagnostic features. This variability in age thresholds likely reflects differences in international guidelines: North American recommendations extend the diagnosis up to 24 months of age [23, 24], whereas European guidelines, including the Italian and French ones, set the limit at 12 months [3, 25].

An earlier Italian survey conducted at a national immunology and allergy congress in 2021 also reported inconsistencies in the diagnosis of bronchiolitis [26]. Such variability is not unique to Italy; a retrospective study in the USA found that 63% of infants with bronchiolitis were misclassified [27].

One of the main new findings of this survey is that the diagnostic heterogeneity was associated with treatment decisions. Almost one-third of respondents reported prescribing oral or inhaled corticosteroids and bronchodilators, despite updated Italian guidelines explicitly discouraging their use [28]. Prescribing practices not supported by current guidelines for bronchiolitis have previously been documented in hospital settings across several countries [20, 29, 30], but this survey provides the first detailed account of such practices in primary care paediatrics in Italy. Our results suggest that misclassification of bronchiolitis may be a contributing factor to inappropriate treatment. Paediatricians who considered an older age threshold were more likely to prescribe antibiotics. Those who considered wheezing as a diagnostic feature were more likely to prescribe bronchodilators and steroids, as were those who did not adhere to an age limit for diagnosis. We speculate that non-guideline-recommended may be, at least in part, due to inaccurate diagnoses, and could potentially be reduced with better diagnostic standardization.

Another heterogeneous finding was the seasonality considered by primary care paediatricians when diagnosing bronchiolitis: 20.9% limited the diagnosis to the epidemic period of respiratory viruses, typically from November to April in Italy [31], 39.9% included spring, and 39.2% considered bronchiolitis a possible diagnosis throughout the entire year. During summer and early fall, bronchiolitis cases are infrequent and are typically associated with non-RSV viruses, such as parainfluenza viruses and rhinoviruses [32].

Antibiotics were primarily prescribed in response to poor general condition and the presence of fever. According to available recommendations, antibiotics should be used only in cases of suspected or confirmed bacterial infection [28]. Our findings are consistent with previous studies [33]. Fever has been reported as associated with severe clinical course and as a possible red flag for bacterial infection [34, 35].

Primary care paediatricians reported the greatest challenges in managing bronchiolitis as follows: refraining from prescribing medications (59.5%), difficulty using pulse oximeter probes due to their size being unsuitable for infants’ small fingers (47.1%), and differences in diagnostic and therapeutic approaches between hospital and community settings (46.7%). The psychological pressure to ‘do something’ in response to parental expectations, the limited availability of studies conducted in primary care settings, and the gap between primary and hospital care should be carefully addressed in future guidelines to reduce the heterogeneity in the management of infants with bronchiolitis [21].

The campaign for nirsevimab prophylaxis—co-administered with the influenza vaccine—was the first implemented at the national level, following the previous regional experience in Valle d’Aosta during the 2023/2024 season [36]. It was generally well received: over 96% of paediatricians expressed support, most parents accepted the administration, and the main criticism concerned organizational aspects. A previous study reported high acceptance of nirsevimab in the maternity units in France [37], while a questionnaire found a high rate of intention to accept its administration during pregnancy or to the infant in the UK [38]. Our findings indicate that implementing nirsevimab prophylaxis through primary care services is both feasible and well accepted, suggesting that the campaign could be successfully repeated in future seasons. Increased uptake of nirsevimab may help reduce both the clinical and economic burden of bronchiolitis [39].

This survey provides important insights for healthcare authorities and policymakers aiming to improve the management of one of the most common illnesses in infancy. Although we pre-tested the questionnaire with five paediatricians and observed high intra-rater reproducibility, it remains a pilot tool and has not been formally validated. Furthermore, the survey was conducted exclusively in Lombardy—a region accounting for nearly one-sixth of the national population—because the study group collaborates with the Lombardy section of the Italian Primary Care Paediatrics Society. While this facilitated survey distribution and engagement, the findings may not be fully generalizable to all regions of Italy. The response rate was 28.8%, likely due to the absence of incentives, which are known to increase participation; however, previous studies of paediatrician surveys suggest that such bias is generally minimal and unlikely to substantially affect the overall findings [40].

Despite these limitations, our study offers important insights by focusing on a specific, under-studied population—primary care paediatricians—and describing their approaches to diagnosing, treating, and preventing bronchiolitis. These professionals play a crucial role in early management and family education [28], yet data on their practices are limited compared with hospital-based care.

## Conclusions

This is the first survey examining how primary care paediatricians in Italy manage bronchiolitis. Our findings reveal notable variation in both diagnostic criteria and therapeutic approaches, mirroring discrepancies previously reported in hospital settings. The association between diagnostic inconsistency and inappropriate prescribing (e.g., bronchodilators and steroids) underscores the need for greater diagnostic standardization as a foundation for improving care. Nirsevimab prophylaxis was widely accepted by both clinicians and families, suggesting that annual implementation in the primary care setting is feasible and likely to be effective in reducing bronchiolitis-related morbidity and costs.

## Supplementary Information

Below is the link to the electronic supplementary material.

Supplementary Material 1

Supplementary Material 2

Supplementary Material 3

## Data Availability

The dataset and R analysis scripts used in this study are deposited in Zenodo (DOI: 10.5281/zenodo.17250698) and will be made publicly available upon publication.

## Abbreviations

CI: Confidence Interval
ED: Emergency Department
EMA: European Medicines Agency
OR: Odds Ratio
RSV: Respiratory Syncytial Virus
SICuPP: Società Italiana delle Cure Primarie Pediatriche
VIF: Variance Inflation Factors

## References

[1] Dalziel SR, Haskell L, O’Brien S, Borland ML, Plint AC, Babl FE, et al. Bronchiolitis Lancet. 2022;400:392–406. 35785792 10.1016/S0140-6736(22)01016-9

[2] Sahai S, Bronchiolitis. Pediatr Care Online [Internet]. 2023 [cited 2025 June 9]; Available from: https://publications.aap.org/pediatriccare/article/doi/10.1542/aap.ppcqr.396139/1484/Bronchiolitis.

[3] Baraldi E, Lanari M, Manzoni P, Rossi GA, Vandini S, Rimini A, et al. Inter-society consensus document on treatment and prevention of bronchiolitis in newborns and infants. Ital J Pediatr. 2014;40:65. 25344148 10.1186/1824-7288-40-65 PMC4364570

[4] GBD 2021 Lower Respiratory Infections and Antimicrobial Resistance Collaborators. Global, regional, and National incidence and mortality burden of non-COVID-19 lower respiratory infections and aetiologies, 1990–2021: a systematic analysis from the global burden of disease study 2021. Lancet Infect Dis. 2024;24:974–1002. 38636536 10.1016/S1473-3099(24)00176-2 PMC11339187

[5] Milani GP, Ronchi A, Agostoni C, Marchisio P, Chidini G, Pesenti N, et al. Long-lasting effects of COVID-19 pandemic on hospitalizations and severity of bronchiolitis. Eur J Pediatr. 2024;183:1751–8. 38236404 10.1007/s00431-023-05395-1 PMC11001736

[6] Hasegawa K, Tsugawa Y, Brown DFM, Mansbach JM, Camargo CA. Trends in bronchiolitis hospitalizations in the united States, 2000–2009. Pediatrics. 2013;132:28–36. 23733801 10.1542/peds.2012-3877 PMC3691534

[7] Griffin MP, Yuan Y, Takas T, Domachowske JB, Madhi SA, Manzoni P, et al. Single-Dose nirsevimab for prevention of RSV in preterm infants. N Engl J Med. 2020;383:415–25. 32726528 10.1056/NEJMoa1913556

[8] Keam SJ, Nirsevimab. First Approval Drugs. 2023;83:181–7. 36577878 10.1007/s40265-022-01829-6

[9] Riccò M, Cascio A, Corrado S, Bottazzoli M, Marchesi F, Gili R et al. Impact of nirsevimab immunization on pediatric hospitalization rates: a systematic review and meta-analysis (2024). Vaccines. 2024;12:640. 10.3390/vaccines12060640 PMC11209424 38932369

[10] Andina Martínez D, Claret Teruel G, Gijón Mediavilla M, Cámara Otegui A, Baños López L, De Miguel Lavisier B, et al. Nirsevimab and acute bronchiolitis episodes in pediatric emergency departments. Pediatrics. 2024;154:e2024066584. 39257372 10.1542/peds.2024-066584

[11] Villani A, Antilici L, Musolino AMC, Merola A, Perno CF, Raponi M, et al. RSV bronchiolitis: a disease only for those who do not receive prophylaxis. Eur J Pediatr. 2025;184:437. 40555916 10.1007/s00431-025-06275-6

[12] Lavagno C, Milani GP, Uestuener P, Simonetti GD, Casaulta C, Bianchetti MG, et al. Hyponatremia in children with acute respiratory infections: A reappraisal. Pediatr Pulmonol. 2017;52:962–7. 28267276 10.1002/ppul.23671

[13] Milani GP, Plebani AM, Arturi E, Brusa D, Esposito S, Dell’Era L, et al. Using a high-flow nasal cannula provided superior results to low-flow oxygen delivery in moderate to severe bronchiolitis. Acta Paediatr Oslo nor 1992. 2016;105:e368–372. 10.1111/apa.13444 27102726

[14] Gong C, Byczkowski T, McAneney C, Goyal MK, Florin TA. Emergency department management of bronchiolitis in the united States. Pediatr Emerg Care. 2019;35:323–9. 28441240 10.1097/PEC.0000000000001145 PMC5654708

[15] Vila J, Lera E, Peremiquel-Trillas P, Martínez L, Barceló I, Andrés C, et al. Management of hospitalized respiratory syncytial virus bronchiolitis in the pediatric ward in spain: assessing the impact of a new clinical practice protocol. Paediatr Drugs. 2022;24:63–71. 34936054 10.1007/s40272-021-00488-6

[16] Kirolos A, Manti S, Blacow R, Tse G, Wilson T, Lister M, et al. A systematic review of clinical practice guidelines for the diagnosis and management of bronchiolitis. J Infect Dis. 2020;222:S672–9. 31541233 10.1093/infdis/jiz240

[17] Lombardia - SICUPP [Internet]. SICUPP. 2025 [cited 2025 May 9]. [Internet]. Available from: https://sicupp.org/la-societa/sicupp-regionale/lombardia/.

[18] Redazione. La variazione del numero di minori in Lombardia [Internet]. Openpolis. Fondazione openpolis; 2021 [cited 2025 May 9]. [Internet]. Available from: https://www.openpolis.it/esercizi/la-variazione-del-numero-di-minori-in-lombardia/.

[19] SISAC - Struttura Interregionale Sanitari Convenzionati [Internet]. Sisac.info. 2024 [cited 2025 May 9]. [Internet]. Available from: https://www.sisac.info/ActionPagina_296.do.

[20] Shapiro DJ, Bourgeois FT, Fine AM, Hersh AL, Coon ER, Neuman MI, et al. National patterns of outpatient Follow-Up visits after emergency care for acute bronchiolitis. JAMA Netw Open. 2023;6:e2340082. 37889492 10.1001/jamanetworkopen.2023.40082 PMC10611989

[21] Porcaro F, Cutrera R, Vittucci AC, Villani A. Bronchiolitis guidelines: what about the Italian situation in a primary care setting? Ital J Pediatr. 2023;49:123. 37726761 10.1186/s13052-023-01527-3 PMC10510229

[22] World Medical Association. WMA Declaration of Helsinki – Ethical Principles for Medical Research Involving Human Participants [Internet]. Wma.net. WMA - the World Medical Association-WMA Declaration of Helsinki – Ethical Principles for Medical Research Involving Human Participants. 2024. [Internet]. Available from: https://www.wma.net/policies-post/wma-declaration-of-helsinki/. 10.1001/jama.2024.21972 39425955

[23] Friedman JN, Rieder MJ, Walton JM, Canadian Paediatric Society, Acute Care Committee. Drug therapy and hazardous substances Committee. Bronchiolitis: recommendations for diagnosis, monitoring and management of children one to 24 months of age. Paediatr Child Health. 2014;19:485–98. 25414585 10.1093/pch/19.9.485 PMC4235450

[24] COMMITTEE ON INFECTIOUS DISEASES AND BRONCHIOLITIS GUIDELINES COMMITTEE, Brady MT, Byington CL, Davies HD, Edwards KM, Jackson MA, et al. Updated guidance for Palivizumab prophylaxis among infants and young children at increased risk of hospitalization for respiratory syncytial virus infection. Pediatrics. 2014;134:415–20. 25070315 10.1542/peds.2014-1665

[25] Milési C, Baudin F, Durand P, Emeriaud G, Essouri S, Pouyau R, et al. Clinical practice guidelines: management of severe bronchiolitis in infants under 12 months old admitted to a pediatric critical care unit. Intensive Care Med. 2023;49:5–25. 36592200 10.1007/s00134-022-06918-4

[26] Manti S, Licari A, Brambilla I, Caffarelli C, Calvani M, Cardinale F, et al. Agreements and controversies of National guidelines for bronchiolitis: results from an Italian survey. Immun Inflamm Dis. 2021;9:1229–36. 34677899 10.1002/iid3.451 PMC8589388

[27] Megalaa R, Perez GF, Kilaikode-Cheruveettara S, Kotwal N, Rodriguez-Martinez CE, Nino G. Clinical definition of respiratory viral infections in young children and potential bronchiolitis misclassification. J Investig Med Off Publ Am Fed Clin Res. 2018;66:46–51. 10.1136/jim-2017-000491 PMC5916841 28947640

[28] Manti S, Staiano A, Orfeo L, Midulla F, Marseglia GL, Ghizzi C, et al. UPDATE – 2022 Italian guidelines on the management of bronchiolitis in infants. Ital J Pediatr. 2023;49:19. 36765418 10.1186/s13052-022-01392-6 PMC9912214

[29] Shanahan KH, Monuteaux MC, Nagler J, Bachur RG. Early use of bronchodilators and outcomes in bronchiolitis. Pediatrics. 2021;148:e2020040394. 34230092 10.1542/peds.2020-040394

[30] Lirette M-P, Kuppermann N, Finkelstein Y, Zemek R, Plint AC, Florin TA, et al. International variation in evidence-based emergency department management of bronchiolitis: a retrospective cohort study. BMJ Open. 2022;12:e059784. 36600373 10.1136/bmjopen-2021-059784 PMC9730363

[31] Azzari C, Baraldi E, Bonanni P, Bozzola E, Coscia A, Lanari M, et al. Epidemiology and prevention of respiratory syncytial virus infections in children in Italy. Ital J Pediatr. 2021;47:198. 34600591 10.1186/s13052-021-01148-8 PMC8487331

[32] Bronchiolitis E. 2012 [cited 2025 July 8]. pp. 231–235.e4. Available from: https://linkinghub.elsevier.com/retrieve/pii/B9781437727029000337.

[33] Zipursky A, Kuppermann N, Finkelstein Y, Zemek R, Plint AC, Babl FE, et al. International practice patterns of antibiotic therapy and laboratory testing in bronchiolitis. Pediatrics. 2020;146:e20193684. 32661190 10.1542/peds.2019-3684

[34] El-Radhi AS, Barry W, Patel S. Association of fever and severe clinical course in bronchiolitis. Arch Dis Child. 1999;81:231–4. 10451396 10.1136/adc.81.3.231 PMC1718060

[35] Elmore D, Yaslam B, Putty K, Magrane T, Abadir A, Bhatt S, et al. Is fever a red flag for bacterial pneumonia in children with viral bronchiolitis? Glob Pediatr Health. 2019;6:2333794X19868660. 31431903 10.1177/2333794X19868660 PMC6686317

[36] Consolati A, Farinelli M, Serravalle P, Rollandin C, Apprato L, Esposito S, et al. Safety and efficacy of nirsevimab in a universal prevention program of respiratory syncytial virus bronchiolitis in newborns and infants in the first year of life in the Valle d’aosta Region, Italy, in the 2023–2024 epidemic season. Vaccines. 2024;12:549. 38793800 10.3390/vaccines12050549 PMC11125727

[37] Ocana de Sentuary C, Testard C, Lagrée M, Leroy M, Gasnier L, Enes-Dias A, et al. Acceptance and safety of the RSV-preventive treatment of newborns with nirsevimab in the maternity department: a prospective longitudinal cohort study in France. EClinicalMedicine. 2025;79:102986. 39726670 10.1016/j.eclinm.2024.102986 PMC11669793

[38] Paulson S, Munro APS, Cathie K, Bedford H, Jones CE. Protecting against respiratory syncytial virus: an online questionnaire study exploring UK parents’ acceptability of vaccination in pregnancy or monoclonal antibody administration for infants. Pediatr Infect Dis J. 2025;44:S158–61. 39951096 10.1097/INF.0000000000004632

[39] Kieffer A, Beuvelet M, Sardesai A, Musci R, Milev S, Roiz J, et al. Expected impact of universal immunization with nirsevimab against RSV-Related outcomes and costs among all US infants in their first RSV season: A static model. J Infect Dis. 2022;226:S282–92. 35968866 10.1093/infdis/jiac216 PMC9377043

[40] Cull WL, O’Connor KG, Sharp S, Tang SS. Response rates and response bias for 50 surveys of pediatricians. Health Serv Res. 2005;40:213–26. 15663710 10.1111/j.1475-6773.2005.00350.x PMC1361134

