# Supplementary material for: Diagnosis, management, and prevention of bronchiolitis in primary care: a survey of Italian family paediatricians
Source: Ital J Pediatr. 2025 Nov 19;51:305. doi: 10.1186/s13052-025-02152-y (PMC12628587; doi:10.1186/s13052-025-02152-y)
Supplement: Supplementary file 1 — Supplementary Material 1 [file 13052_2025_2152_MOESM1_ESM.docx]

**Diagnosis, Management, and Prevention of Bronchiolitis in Primary Care: A Survey of Italian Family Pediatricians**

**Supplementary materials**

**Appendix A**

Final version of the survey and its English translation

| **ITALIAN VERSION** | **ENGLISH TRANSLATION** |
| --- | --- |
| **Da quanti anni eserciti la professione di pediatra di famiglia** | **How many years have you been practicing as a family pediatrician?** |
| <10 anni | <10 years |
| 10-20 anni | 10–20 years |
| >20 anni | >20 years |
| **Qual è il limite superiore di età che consideri per diagnosticare la bronchiolite?** | **What is the upper age limit you consider for diagnosing bronchiolitis?** |
| ≤6 mesi | ≤6 months |
| ≤12 mesi | ≤12 months |
| ≤24 mesi | ≤24 months |
| ≤36 mesi | ≤36 months |
| Non considero nessun limite superiore di età | I do not consider any upper age limit |
| **Posto il cut-off di età considerato adeguato, in quali casi effettui la diagnosi di bronchiolite?** | **Given the considered appropriate age cut-off, in which cases do you diagnose bronchiolitis?** |
| Prodromi di infezione delle alte vie aeree e successivi rumori diffusi all’auscultazione delle basse vie aeree, indipendentemente dalla loro tipologia | Prodromes of upper respiratory tract infection and subsequent diffuse sounds on auscultation of the lower airways, regardless of their type |
| Prodromi di infezione delle alte vie aeree e successivi segni di distress respiratorio con gemiti e/o sibili all’auscultazione delle basse vie aeree | Prodromes of upper respiratory tract infection and subsequent signs of respiratory distress with grunting and/or wheezing on auscultation of the lower airways |
| Prodromi di infezione delle alte vie aeree e successivi segni di distress respiratorio con rumori umidi diffusi all’auscultazione delle basse vie aeree (per esempio rantoli o crepitii) | Prodromes of upper respiratory tract infection and subsequent signs of respiratory distress with diffuse moist sounds on auscultation of the lower airways (e.g., crackles or rales) |
| **Posti i criteri selezionati, consideri la diagnosi di bronchiolite in quali periodi dell'anno?** | **Given the selected criteria, during which periods of the year do you consider the diagnosis of bronchiolitis?** |
| Solo durante il periodo epidemico dei virus respiratori tipici di bronchiolite (autunno-inverno) | Only during the epidemic period of respiratory viruses typical of bronchiolitis (autumn-winter) |
| Solo durante il periodo epidemico dei virus respiratori tipici di bronchiolite (autunno-inverno) e in primavera | Only during the epidemic period of respiratory viruses typical of bronchiolitis (autumn-winter) and in spring |
| Durante tutto l’anno | Throughout the entire year |
| **Misuri la frequenza respiratoria nei pazienti in cui effettui diagnosi di bronchiolite?** | **Do you measure respiratory rate in patients you diagnose with bronchiolitis?** |
| Mai | Never |
| Solo in casi di grave distress respiratorio | Only in cases of severe respiratory distress |
| Sempre | Always |
| **Utilizzi la saturimetria per monitorare i pazienti in cui effettui diagnosi di bronchiolite?** | **Do you use pulse oximetry to monitor patients diagnosed with bronchiolitis?** |
| Mai | Never |
| In casi selezionati in base alla disponibilità di una sonda del saturimetro adeguata al dito del bambino/a | In selected cases based on the availability of a pulse oximeter probe suitable for the child's finger |
| Solo in casi di grave distress respiratorio | Only in cases of severe respiratory distress |
| Sempre | Always |
| **Effettui dei test rapidi per la diagnosi eziologica della bronchiolite?** | **Do you perform rapid tests for the etiological diagnosis of bronchiolitis?** |
| Mai | Never |
| Saltuariamente | Occasionally |
| Sempre | Always |
| **Quali test effettui?** | **Which tests do you perform?** |
| Per la ricerca del virus respiratorio sinciziale | To detect respiratory syncytial virus |
| Per la ricerca del virus respiratorio sinciziale e dell’influenza | To detect respiratory syncytial virus and influenza |
| Per la ricerca del virus respiratorio sinciziale, dell’influenza, del SARS-CoV-2 | To detect respiratory syncytial virus, influenza, and SARS-CoV-2 |
| Per la ricerca del virus respiratorio sinciziale, dell’influenza, del SARS-CoV-2 e altri virus/batteri | To detect respiratory syncytial virus, influenza, SARS-CoV-2, and other viruses/bacteria |
|  |  |
| **Nella gestione ambulatoriale della bronchiolite quali indicazioni routinariamente fornisci ai genitori?** | **In the outpatient management of bronchiolitis, what routine advice do you give to parents?** |
| **Monitoraggio dello stato generale/benessere** | **Monitoring of general condition/well-being** |
| No | No |
| Si | Yes |
| **Sorveglianza dell’apporto nutrizionale/idratazione** | **Monitoring of nutritional/hydration intake** |
| No | No |
| Si | Yes |
| **Uso di broncodilatatori** | **Use of bronchodilators** |
| No | No |
| Si | Yes |
| **Uso di steroidi inalatori o per bocca** | **Use of inhaled or oral steroids** |
| No | No |
| Si | Yes |
| **Uso di aerosol con ipertonica** | **Use of hypertonic saline aerosol** |
| No | No |
| Si | Yes |
| **Lavaggi nasali** | **Nasal irrigations** |
| No | No |
| Si | Yes |
| **Altro** | **Other** |
| No | No |
| Si | Yes |
| **Specificare quale altre indicazioni fornisci ai genitori** | **Specify what other indications you provide to parents** |
| **Quale broncodilatatore prescrivi solitamente?** | **Which bronchodilator do you usually prescribe?** |
| Salbutamolo | Salbutamol |
| Sia salbutamolo che Ipratropio bromuro | Both salbutamol and ipratropium bromide |
|  |  |
| **In quali di queste circostanze decidi di inviare un paziente con bronchiolite in ospedale?** | **Under which of the following circumstances do you decide to refer a patient with bronchiolitis to the hospital?** |
| **Saturazione dell'ossigeno in aria ambiente <90%** | **Oxygen saturation in room air <90%** |
| No | No |
| Si | Yes |
| **Saturazione dell'ossigeno in aria ambiente <92%** | **Oxygen saturation in room air <92%** |
| No | No |
| Si | Yes |
| **Episodi di apnee riferiti dal genitore e/o riscontrabili durante la visita** | **Episodes of apnea reported by the parent and/or observed during the visit** |
| No | No |
| Si | Yes |
| **Importante distress respiratorio indipendentemente da un dato di saturazione dell’ossigeno normale** | **Significant respiratory distress regardless of normal oxygen saturation values** |
| No | No |
| Si | Yes |
| **Disidratazione e/o apporto nutrizionale <50% nelle 24 ore precedenti** | **Dehydration and/or nutritional intake <50% in the previous 24 hours** |
| No | No |
| Si | Yes |
| **Età <1 mese** | **Age <1 month** |
| No | No |
| Si | Yes |
| **Età <3 mesi** | **Age <3 months** |
| No | No |
| Si | Yes |
| **Prematurità (<35 settimane di età gestazionale) / patologia cronica predisponente a bronchiolite severa** | **Prematurity (<35 weeks of gestational age) / chronic condition predisposing to severe bronchiolitis** |
| No | No |
| Si | Yes |
| **Scarsa compliance familiare** | **Low family compiance** |
| No | No |
| Si | Yes |
| **Febbre >39°C** | **Fever >39°C** |
| No | No |
| Si | Yes |
| **Febbre che non risponde all’antipiretico** | **Fever that does not respond to antipyretics** |
| No | No |
| Si | Yes |
|  |  |
| **Raccomandi l'uso di fisioterapia respiratoria nei pazienti con bronchiolite?** | **Do you recommend the use of respiratory physiotherapy in patients with bronchiolitis?** |
| Mai | Never |
| Saltuariamente | Occasionally |
| Sempre | Always |
| **Raccomandi l'uso di trattamenti osteopatici nei pazienti con bronchiolite?** | **Do you recommend the use of osteopathic treatments in patients with bronchiolitis?** |
| Mai | Never |
| Saltuariamente | Occasionally |
| Sempre | Always |
|  |  |
| **In quali di questi casi prescrivi solitamente antibiotici a scopo profilattico nei pazienti con bronchiolite?** | **In which of the following cases do you usually prescribe prophylactic antibiotics in patients with bronchiolitis?** |
| **Sempre** | **Always** |
| No | No |
| Si | Yes |
| **Nei pazienti con <1 mese di età** | **In patients younger than 1 month of age** |
| No | No |
| Si | Yes |
| **Nei pazienti con <3 mesi di età** | **In patients younger than 3 months of age** |
| No | No |
| Si | Yes |
| **In caso di febbre** | **In case of fever** |
| No | No |
| Si | Yes |
| **In caso di compromissione delle condizioni generali** | **In case of poor general condition** |
| No | No |
| Si | Yes |
| **In caso di ridotto apporto nutrizionale** | **In case of reduced nutritional intake** |
| No | No |
| Si | Yes |
| **In caso di distress respiratorio importante** | **In case of severe respiratory distress** |
| No | No |
| Si | Yes |
| **Mai** | **Never** |
| No | No |
| Si | Yes |
|  |  |
| **Quale di queste ritieni sia la maggior difficoltà nella gestione della bronchiolite, rispetto alle linee guida?** | **What do you consider to be the greatest challenge in managing bronchiolitis in accordance with clinical guidelines?** |
| **Difficoltà nella diagnosi** | **Difficulty in diagnosis** |
| No | No |
| Si | Yes |
| **Difficoltà nel non prescrivere farmaci** | **Difficulty refraining from prescribing medications** |
| No | No |
| Si | Yes |
| **Difficoltà nel seguire il paziente sul territorio** | **Difficulty in following up with the patient in the community** |
| No | No |
| Si | Yes |
| **Differenti approcci diagnostico terapeutici territorio ospedale** | **Different diagnostic and therapeutic approaches between community and hospital settings** |
| No | No |
| Si | Yes |
| **Difficoltà di utilizzo della sonda del saturimetro, spesso non adeguato al dito del bambino molto piccolo** | **Difficulty using the pulse oximeter probe, often not suitable for the finger of very small infants** |
| No | No |
| Si | Yes |
| **Altro** | **Other** |
| No | No |
| Si | Yes |
| **Specificare quale altra maggior difficoltà ritrovi nella gestione della bronchiolite, rispetto alle linee guida?** | **Specify what other major difficulty you encounter in managing bronchiolitis, compared to the guidelines?** |
|  |  |
| **Cosa pensi della nuova profilassi con Nirsevimab per l’infezione da virus respiratorio sinciziale?** | **What do you think about the new prophylaxis with Nirsevimab for RSV infection?** |
| Favorevole in tutti neonati/lattanti | Favorable in all newborns/infants |
| Favorevole solo in alcuni casi | Favorable only in selected cases |
| Non favorevole | Not favorable |
| **Hai partecipato alla profilassi con Nirsevimab nel tuo ambulatorio?** | **Have you participated in prophylaxis with Nirsevimab in your clinic?** |
| No | No |
| Si | Yes |
| **La maggior parte dei genitori/caregivers come si è posta rispetto all'immunoprofilassi con nirsevimab?** | **How have most parents/caregivers responded to immunoprophylaxis with Nirsevimab?** |
| Ha accettato subito di eseguire la profilassi | They immediately agreed to undergo the prophylaxis |
| Ha inizialmente rifiutato/opposto resistenza alla profilassi ma poi ha accettato | They initially refused/opposed the prophylaxis but later accepted |
| Ha rifiutato di eseguire la profilassi | He/She refused to undergo the prophylaxis |
| **Come descriveresti la tua esperienza relativa all’immunoprofilassi?** | **How would you describe your experience with immunoprophylaxis?** |
| Molto negativa | Very negative |
| Negativa | Negative |
| Positiva | Positive |
| Molto positiva | Very positive |
| **Saresti disposto a ripeterla nella futura stagione?** | **Would you be willing to repeat it in the upcoming season?** |
| No | No |
| Non so | I don’t know |
| Sì, ma solo a condizioni organizzative migliori | Yes, but only under better organizational conditions |
| Sì | Yes |
| **Saresti disposto ad eseguirla nella futura stagione?** | **Would you be willing to carry it out in the upcoming season?** |
| No | No |
| Non so | I don’t know |
| Sì, ma solo a condizioni organizzative migliori | Yes, but only under better organizational conditions |
| Sì | Yes |

**Appendix B**

Diagnostic assessment of multivariable logistic regression models for antibiotic, bronchodilator, and steroid prescribing

| **Model (Outcome)** | **Max VIF** | **Hosmer Lemeshow χ² (df), p** | **Calibration intercept (95% CI)** | **Calibration slope (95% CI)** | **Notes** |
| --- | --- | --- | --- | --- | --- |
| Antibiotic use | 1.01 | 2.35 (5), p = 0.80 | 0.00 (–0.24 to 0.23) | 1.00 (0.50 to 1.56) | Good fit, no multicollinearity |
| Bronchodilator use | 1.01 | 4.96 (6), p = 0.55 | 0.00 (–0.24 to 0.23) | 1.00 (0.40 to 1.63) | Good fit, no multicollinearity |
| Steroid use | 1.01 | 5.87 (6), p = 0.44 | –0.238 (–0.486 to 0.002) | 1.00 (0.42 to 1.70) | Good fit, no multicollinearity |
