# Supplementary material for: Diagnosis, management, and prevention of bronchiolitis in primary care: a survey of Italian family paediatricians
Source: Ital J Pediatr. 2025 Nov 19;51:305. doi: 10.1186/s13052-025-02152-y (PMC12628587; doi:10.1186/s13052-025-02152-y)
Supplement: Supplementary file 2 — Supplementary Material 2 [file 13052_2025_2152_MOESM2_ESM.docx]

**RLOGS_Bronchidilators_Bronchiolitis_Diagnosis (age and symptoms)**

> summary(GLM.4)

Call:

glm(formula = Nella.gestione.ambulatoriale.della.bronchiolite.quali.indicazioni.routinariamente.fornisci.ai.genitori...Uso.di.broncodilatatori. ~

Qual.è.il.limite.superiore.di.età.che.consideri.per.diagnosticare.la.bronchiolite.. +

Posto.il.cut.off.di.età.considerato.adeguato..in.quali.casi.effettui.la.diagnosi.di.bronchiolite.,

family = binomial(logit), data = SP)

Coefficients:

Estimate

(Intercept) -0.30135

Qual.è.il.limite.superiore.di.età.che.consideri.per.diagnosticare.la.bronchiolite..[T.≤24 mesi] 0.02012

Qual.è.il.limite.superiore.di.età.che.consideri.per.diagnosticare.la.bronchiolite..[T.≤36 mesi] 0.29646

Qual.è.il.limite.superiore.di.età.che.consideri.per.diagnosticare.la.bronchiolite..[T.≤6 mesi] 0.79303

Qual.è.il.limite.superiore.di.età.che.consideri.per.diagnosticare.la.bronchiolite..[T.Non considero nessun limite superiore di età] 0.40073

Posto.il.cut.off.di.età.considerato.adeguato..in.quali.casi.effettui.la.diagnosi.di.bronchiolite.[T.Prodromi di infezione delle alte vie aeree e successivi rumori diffusi all’auscultazione delle basse vie aeree, indipendentemente dalla loro tipologia] -0.95849

Posto.il.cut.off.di.età.considerato.adeguato..in.quali.casi.effettui.la.diagnosi.di.bronchiolite.[T.Prodromi di infezione delle alte vie aeree e successivi segni di distress respiratorio con rumori umidi diffusi all’auscultazione delle basse vie aeree (per esempio rantoli o crepitii)] -0.37576

Std. Error

(Intercept) 0.22065

Qual.è.il.limite.superiore.di.età.che.consideri.per.diagnosticare.la.bronchiolite..[T.≤24 mesi] 0.26216

Qual.è.il.limite.superiore.di.età.che.consideri.per.diagnosticare.la.bronchiolite..[T.≤36 mesi] 0.61396

Qual.è.il.limite.superiore.di.età.che.consideri.per.diagnosticare.la.bronchiolite..[T.≤6 mesi] 0.54282

Qual.è.il.limite.superiore.di.età.che.consideri.per.diagnosticare.la.bronchiolite..[T.Non considero nessun limite superiore di età] 0.52559

Posto.il.cut.off.di.età.considerato.adeguato..in.quali.casi.effettui.la.diagnosi.di.bronchiolite.[T.Prodromi di infezione delle alte vie aeree e successivi rumori diffusi all’auscultazione delle basse vie aeree, indipendentemente dalla loro tipologia] 0.36516

Posto.il.cut.off.di.età.considerato.adeguato..in.quali.casi.effettui.la.diagnosi.di.bronchiolite.[T.Prodromi di infezione delle alte vie aeree e successivi segni di distress respiratorio con rumori umidi diffusi all’auscultazione delle basse vie aeree (per esempio rantoli o crepitii)] 0.26252

z value

(Intercept) -1.366

Qual.è.il.limite.superiore.di.età.che.consideri.per.diagnosticare.la.bronchiolite..[T.≤24 mesi] 0.077

Qual.è.il.limite.superiore.di.età.che.consideri.per.diagnosticare.la.bronchiolite..[T.≤36 mesi] 0.483

Qual.è.il.limite.superiore.di.età.che.consideri.per.diagnosticare.la.bronchiolite..[T.≤6 mesi] 1.461

Qual.è.il.limite.superiore.di.età.che.consideri.per.diagnosticare.la.bronchiolite..[T.Non considero nessun limite superiore di età] 0.762

Posto.il.cut.off.di.età.considerato.adeguato..in.quali.casi.effettui.la.diagnosi.di.bronchiolite.[T.Prodromi di infezione delle alte vie aeree e successivi rumori diffusi all’auscultazione delle basse vie aeree, indipendentemente dalla loro tipologia] -2.625

Posto.il.cut.off.di.età.considerato.adeguato..in.quali.casi.effettui.la.diagnosi.di.bronchiolite.[T.Prodromi di infezione delle alte vie aeree e successivi segni di distress respiratorio con rumori umidi diffusi all’auscultazione delle basse vie aeree (per esempio rantoli o crepitii)] -1.431

Pr(>|z|)

(Intercept) 0.17202

Qual.è.il.limite.superiore.di.età.che.consideri.per.diagnosticare.la.bronchiolite..[T.≤24 mesi] 0.93881

Qual.è.il.limite.superiore.di.età.che.consideri.per.diagnosticare.la.bronchiolite..[T.≤36 mesi] 0.62919

Qual.è.il.limite.superiore.di.età.che.consideri.per.diagnosticare.la.bronchiolite..[T.≤6 mesi] 0.14403

Qual.è.il.limite.superiore.di.età.che.consideri.per.diagnosticare.la.bronchiolite..[T.Non considero nessun limite superiore di età] 0.44580

Posto.il.cut.off.di.età.considerato.adeguato..in.quali.casi.effettui.la.diagnosi.di.bronchiolite.[T.Prodromi di infezione delle alte vie aeree e successivi rumori diffusi all’auscultazione delle basse vie aeree, indipendentemente dalla loro tipologia] 0.00867

Posto.il.cut.off.di.età.considerato.adeguato..in.quali.casi.effettui.la.diagnosi.di.bronchiolite.[T.Prodromi di infezione delle alte vie aeree e successivi segni di distress respiratorio con rumori umidi diffusi all’auscultazione delle basse vie aeree (per esempio rantoli o crepitii)] 0.15233

(Intercept)

Qual.è.il.limite.superiore.di.età.che.consideri.per.diagnosticare.la.bronchiolite..[T.≤24 mesi]

Qual.è.il.limite.superiore.di.età.che.consideri.per.diagnosticare.la.bronchiolite..[T.≤36 mesi]

Qual.è.il.limite.superiore.di.età.che.consideri.per.diagnosticare.la.bronchiolite..[T.≤6 mesi]

Qual.è.il.limite.superiore.di.età.che.consideri.per.diagnosticare.la.bronchiolite..[T.Non considero nessun limite superiore di età]

Posto.il.cut.off.di.età.considerato.adeguato..in.quali.casi.effettui.la.diagnosi.di.bronchiolite.[T.Prodromi di infezione delle alte vie aeree e successivi rumori diffusi all’auscultazione delle basse vie aeree, indipendentemente dalla loro tipologia] **

Posto.il.cut.off.di.età.considerato.adeguato..in.quali.casi.effettui.la.diagnosi.di.bronchiolite.[T.Prodromi di infezione delle alte vie aeree e successivi segni di distress respiratorio con rumori umidi diffusi all’auscultazione delle basse vie aeree (per esempio rantoli o crepitii)]

---

Signif. codes: 0 '***' 0.001 '**' 0.01 '*' 0.05 '.' 0.1 ' ' 1

(Dispersion parameter for binomial family taken to be 1)

Null deviance: 405.13 on 305 degrees of freedom

Residual deviance: 394.10 on 299 degrees of freedom

AIC: 408.1

Number of Fisher Scoring iterations: 4

> exp(coef(GLM.4)) # Exponentiated coefficients ("odds ratios")

(Intercept)

0.7398197

Qual.è.il.limite.superiore.di.età.che.consideri.per.diagnosticare.la.bronchiolite..[T.≤24 mesi]

1.0203284

Qual.è.il.limite.superiore.di.età.che.consideri.per.diagnosticare.la.bronchiolite..[T.≤36 mesi]

1.3450882

Qual.è.il.limite.superiore.di.età.che.consideri.per.diagnosticare.la.bronchiolite..[T.≤6 mesi]

2.2100830

Qual.è.il.limite.superiore.di.età.che.consideri.per.diagnosticare.la.bronchiolite..[T.Non considero nessun limite superiore di età]

1.4929128

Posto.il.cut.off.di.età.considerato.adeguato..in.quali.casi.effettui.la.diagnosi.di.bronchiolite.[T.Prodromi di infezione delle alte vie aeree e successivi rumori diffusi all’auscultazione delle basse vie aeree, indipendentemente dalla loro tipologia]

0.3834721

Posto.il.cut.off.di.età.considerato.adeguato..in.quali.casi.effettui.la.diagnosi.di.bronchiolite.[T.Prodromi di infezione delle alte vie aeree e successivi segni di distress respiratorio con rumori umidi diffusi all’auscultazione delle basse vie aeree (per esempio rantoli o crepitii)]

0.6867637

> Confint(GLM.4, level=0.95, type="LR")

Estimate

(Intercept) -0.30134871

Qual.è.il.limite.superiore.di.età.che.consideri.per.diagnosticare.la.bronchiolite..[T.≤24 mesi] 0.02012449

Qual.è.il.limite.superiore.di.età.che.consideri.per.diagnosticare.la.bronchiolite..[T.≤36 mesi] 0.29645962

Qual.è.il.limite.superiore.di.età.che.consideri.per.diagnosticare.la.bronchiolite..[T.≤6 mesi] 0.79303008

Qual.è.il.limite.superiore.di.età.che.consideri.per.diagnosticare.la.bronchiolite..[T.Non considero nessun limite superiore di età] 0.40072909

Posto.il.cut.off.di.età.considerato.adeguato..in.quali.casi.effettui.la.diagnosi.di.bronchiolite.[T.Prodromi di infezione delle alte vie aeree e successivi rumori diffusi all’auscultazione delle basse vie aeree, indipendentemente dalla loro tipologia] -0.95848833

Posto.il.cut.off.di.età.considerato.adeguato..in.quali.casi.effettui.la.diagnosi.di.bronchiolite.[T.Prodromi di infezione delle alte vie aeree e successivi segni di distress respiratorio con rumori umidi diffusi all’auscultazione delle basse vie aeree (per esempio rantoli o crepitii)] -0.37576495

2.5 %

(Intercept) -0.7388416

Qual.è.il.limite.superiore.di.età.che.consideri.per.diagnosticare.la.bronchiolite..[T.≤24 mesi] -0.4944112

Qual.è.il.limite.superiore.di.età.che.consideri.per.diagnosticare.la.bronchiolite..[T.≤36 mesi] -0.9716528

Qual.è.il.limite.superiore.di.età.che.consideri.per.diagnosticare.la.bronchiolite..[T.≤6 mesi] -0.2684476

Qual.è.il.limite.superiore.di.età.che.consideri.per.diagnosticare.la.bronchiolite..[T.Non considero nessun limite superiore di età] -0.6513855

Posto.il.cut.off.di.età.considerato.adeguato..in.quali.casi.effettui.la.diagnosi.di.bronchiolite.[T.Prodromi di infezione delle alte vie aeree e successivi rumori diffusi all’auscultazione delle basse vie aeree, indipendentemente dalla loro tipologia] -1.7048871

Posto.il.cut.off.di.età.considerato.adeguato..in.quali.casi.effettui.la.diagnosi.di.bronchiolite.[T.Prodromi di infezione delle alte vie aeree e successivi segni di distress respiratorio con rumori umidi diffusi all’auscultazione delle basse vie aeree (per esempio rantoli o crepitii)] -0.8942188

97.5 %

(Intercept) 0.1286319

Qual.è.il.limite.superiore.di.età.che.consideri.per.diagnosticare.la.bronchiolite..[T.≤24 mesi] 0.5350067

Qual.è.il.limite.superiore.di.età.che.consideri.per.diagnosticare.la.bronchiolite..[T.≤36 mesi] 1.4868102

Qual.è.il.limite.superiore.di.età.che.consideri.per.diagnosticare.la.bronchiolite..[T.≤6 mesi] 1.8927847

Qual.è.il.limite.superiore.di.età.che.consideri.per.diagnosticare.la.bronchiolite..[T.Non considero nessun limite superiore di età] 1.4383741

Posto.il.cut.off.di.età.considerato.adeguato..in.quali.casi.effettui.la.diagnosi.di.bronchiolite.[T.Prodromi di infezione delle alte vie aeree e successivi rumori diffusi all’auscultazione delle basse vie aeree, indipendentemente dalla loro tipologia] -0.2645236

Posto.il.cut.off.di.età.considerato.adeguato..in.quali.casi.effettui.la.diagnosi.di.bronchiolite.[T.Prodromi di infezione delle alte vie aeree e successivi segni di distress respiratorio con rumori umidi diffusi all’auscultazione delle basse vie aeree (per esempio rantoli o crepitii)] 0.1365900

> Confint(GLM.4, level=0.95, type="LR", exponentiate=TRUE)

Exponentiated Coefficients and Confidence Bounds

Estimate

(Intercept) 0.7398197

Qual.è.il.limite.superiore.di.età.che.consideri.per.diagnosticare.la.bronchiolite..[T.≤24 mesi] 1.0203284

Qual.è.il.limite.superiore.di.età.che.consideri.per.diagnosticare.la.bronchiolite..[T.≤36 mesi] 1.3450882

Qual.è.il.limite.superiore.di.età.che.consideri.per.diagnosticare.la.bronchiolite..[T.≤6 mesi] 2.2100830

Qual.è.il.limite.superiore.di.età.che.consideri.per.diagnosticare.la.bronchiolite..[T.Non considero nessun limite superiore di età] 1.4929128

Posto.il.cut.off.di.età.considerato.adeguato..in.quali.casi.effettui.la.diagnosi.di.bronchiolite.[T.Prodromi di infezione delle alte vie aeree e successivi rumori diffusi all’auscultazione delle basse vie aeree, indipendentemente dalla loro tipologia] 0.3834721

Posto.il.cut.off.di.età.considerato.adeguato..in.quali.casi.effettui.la.diagnosi.di.bronchiolite.[T.Prodromi di infezione delle alte vie aeree e successivi segni di distress respiratorio con rumori umidi diffusi all’auscultazione delle basse vie aeree (per esempio rantoli o crepitii)] 0.6867637

2.5 %

(Intercept) 0.4776669

Qual.è.il.limite.superiore.di.età.che.consideri.per.diagnosticare.la.bronchiolite..[T.≤24 mesi] 0.6099300

Qual.è.il.limite.superiore.di.età.che.consideri.per.diagnosticare.la.bronchiolite..[T.≤36 mesi] 0.3784570

Qual.è.il.limite.superiore.di.età.che.consideri.per.diagnosticare.la.bronchiolite..[T.≤6 mesi] 0.7645655

Qual.è.il.limite.superiore.di.età.che.consideri.per.diagnosticare.la.bronchiolite..[T.Non considero nessun limite superiore di età] 0.5213230

Posto.il.cut.off.di.età.considerato.adeguato..in.quali.casi.effettui.la.diagnosi.di.bronchiolite.[T.Prodromi di infezione delle alte vie aeree e successivi rumori diffusi all’auscultazione delle basse vie aeree, indipendentemente dalla loro tipologia] 0.1817929

Posto.il.cut.off.di.età.considerato.adeguato..in.quali.casi.effettui.la.diagnosi.di.bronchiolite.[T.Prodromi di infezione delle alte vie aeree e successivi segni di distress respiratorio con rumori umidi diffusi all’auscultazione delle basse vie aeree (per esempio rantoli o crepitii)] 0.4089269

97.5 %

(Intercept) 1.1372714

Qual.è.il.limite.superiore.di.età.che.consideri.per.diagnosticare.la.bronchiolite..[T.≤24 mesi] 1.7074597

Qual.è.il.limite.superiore.di.età.che.consideri.per.diagnosticare.la.bronchiolite..[T.≤36 mesi] 4.4229648

Qual.è.il.limite.superiore.di.età.che.consideri.per.diagnosticare.la.bronchiolite..[T.≤6 mesi] 6.6378271

Qual.è.il.limite.superiore.di.età.che.consideri.per.diagnosticare.la.bronchiolite..[T.Non considero nessun limite superiore di età] 4.2138388

Posto.il.cut.off.di.età.considerato.adeguato..in.quali.casi.effettui.la.diagnosi.di.bronchiolite.[T.Prodromi di infezione delle alte vie aeree e successivi rumori diffusi all’auscultazione delle basse vie aeree, indipendentemente dalla loro tipologia] 0.7675715

Posto.il.cut.off.di.età.considerato.adeguato..in.quali.casi.effettui.la.diagnosi.di.bronchiolite.[T.Prodromi di infezione delle alte vie aeree e successivi segni di distress respiratorio con rumori umidi diffusi all’auscultazione delle basse vie aeree (per esempio rantoli o crepitii)] 1.1463580

**RLOGS_ANTIBIOTICS (age and symptoms)**

> summary(GLM.2)

Call:

glm(formula = In.quali.di.questi.casi.prescrivi.solitamente.antibiotici.a.scopo.profilattico.nei.pazienti.con.bronchiolite....Mai. ~

Qual.è.il.limite.superiore.di.età.che.consideri.per.diagnosticare.la.bronchiolite.. +

Posto.il.cut.off.di.età.considerato.adeguato..in.quali.casi.effettui.la.diagnosi.di.bronchiolite.,

family = binomial(logit), data = BR)

Coefficients:

Estimate

(Intercept) -0.13617

Qual.è.il.limite.superiore.di.età.che.consideri.per.diagnosticare.la.bronchiolite..[T.≤24 mesi] 0.06973

Qual.è.il.limite.superiore.di.età.che.consideri.per.diagnosticare.la.bronchiolite..[T.≤36 mesi] -2.06640

Qual.è.il.limite.superiore.di.età.che.consideri.per.diagnosticare.la.bronchiolite..[T.≤6 mesi] -0.34117

Qual.è.il.limite.superiore.di.età.che.consideri.per.diagnosticare.la.bronchiolite..[T.Non considero nessun limite superiore di età] -0.43546

Posto.il.cut.off.di.età.considerato.adeguato..in.quali.casi.effettui.la.diagnosi.di.bronchiolite.[T.Prodromi di infezione delle alte vie aeree e successivi rumori diffusi all’auscultazione delle basse vie aeree, indipendentemente dalla loro tipologia] 0.88344

Posto.il.cut.off.di.età.considerato.adeguato..in.quali.casi.effettui.la.diagnosi.di.bronchiolite.[T.Prodromi di infezione delle alte vie aeree e successivi segni di distress respiratorio con rumori umidi diffusi all’auscultazione delle basse vie aeree (per esempio rantoli o crepitii)] 0.36023

Std. Error

(Intercept) 0.21739

Qual.è.il.limite.superiore.di.età.che.consideri.per.diagnosticare.la.bronchiolite..[T.≤24 mesi] 0.25238

Qual.è.il.limite.superiore.di.età.che.consideri.per.diagnosticare.la.bronchiolite..[T.≤36 mesi] 0.80387

Qual.è.il.limite.superiore.di.età.che.consideri.per.diagnosticare.la.bronchiolite..[T.≤6 mesi] 0.53979

Qual.è.il.limite.superiore.di.età.che.consideri.per.diagnosticare.la.bronchiolite..[T.Non considero nessun limite superiore di età] 0.52925

Posto.il.cut.off.di.età.considerato.adeguato..in.quali.casi.effettui.la.diagnosi.di.bronchiolite.[T.Prodromi di infezione delle alte vie aeree e successivi rumori diffusi all’auscultazione delle basse vie aeree, indipendentemente dalla loro tipologia] 0.34371

Posto.il.cut.off.di.età.considerato.adeguato..in.quali.casi.effettui.la.diagnosi.di.bronchiolite.[T.Prodromi di infezione delle alte vie aeree e successivi segni di distress respiratorio con rumori umidi diffusi all’auscultazione delle basse vie aeree (per esempio rantoli o crepitii)] 0.25848

z value

(Intercept) -0.626

Qual.è.il.limite.superiore.di.età.che.consideri.per.diagnosticare.la.bronchiolite..[T.≤24 mesi] 0.276

Qual.è.il.limite.superiore.di.età.che.consideri.per.diagnosticare.la.bronchiolite..[T.≤36 mesi] -2.571

Qual.è.il.limite.superiore.di.età.che.consideri.per.diagnosticare.la.bronchiolite..[T.≤6 mesi] -0.632

Qual.è.il.limite.superiore.di.età.che.consideri.per.diagnosticare.la.bronchiolite..[T.Non considero nessun limite superiore di età] -0.823

Posto.il.cut.off.di.età.considerato.adeguato..in.quali.casi.effettui.la.diagnosi.di.bronchiolite.[T.Prodromi di infezione delle alte vie aeree e successivi rumori diffusi all’auscultazione delle basse vie aeree, indipendentemente dalla loro tipologia] 2.570

Posto.il.cut.off.di.età.considerato.adeguato..in.quali.casi.effettui.la.diagnosi.di.bronchiolite.[T.Prodromi di infezione delle alte vie aeree e successivi segni di distress respiratorio con rumori umidi diffusi all’auscultazione delle basse vie aeree (per esempio rantoli o crepitii)] 1.394

Pr(>|z|)

(Intercept) 0.5311

Qual.è.il.limite.superiore.di.età.che.consideri.per.diagnosticare.la.bronchiolite..[T.≤24 mesi] 0.7823

Qual.è.il.limite.superiore.di.età.che.consideri.per.diagnosticare.la.bronchiolite..[T.≤36 mesi] 0.0102

Qual.è.il.limite.superiore.di.età.che.consideri.per.diagnosticare.la.bronchiolite..[T.≤6 mesi] 0.5274

Qual.è.il.limite.superiore.di.età.che.consideri.per.diagnosticare.la.bronchiolite..[T.Non considero nessun limite superiore di età] 0.4106

Posto.il.cut.off.di.età.considerato.adeguato..in.quali.casi.effettui.la.diagnosi.di.bronchiolite.[T.Prodromi di infezione delle alte vie aeree e successivi rumori diffusi all’auscultazione delle basse vie aeree, indipendentemente dalla loro tipologia] 0.0102

Posto.il.cut.off.di.età.considerato.adeguato..in.quali.casi.effettui.la.diagnosi.di.bronchiolite.[T.Prodromi di infezione delle alte vie aeree e successivi segni di distress respiratorio con rumori umidi diffusi all’auscultazione delle basse vie aeree (per esempio rantoli o crepitii)] 0.1634

(Intercept)

Qual.è.il.limite.superiore.di.età.che.consideri.per.diagnosticare.la.bronchiolite..[T.≤24 mesi]

Qual.è.il.limite.superiore.di.età.che.consideri.per.diagnosticare.la.bronchiolite..[T.≤36 mesi] *

Qual.è.il.limite.superiore.di.età.che.consideri.per.diagnosticare.la.bronchiolite..[T.≤6 mesi]

Qual.è.il.limite.superiore.di.età.che.consideri.per.diagnosticare.la.bronchiolite..[T.Non considero nessun limite superiore di età]

Posto.il.cut.off.di.età.considerato.adeguato..in.quali.casi.effettui.la.diagnosi.di.bronchiolite.[T.Prodromi di infezione delle alte vie aeree e successivi rumori diffusi all’auscultazione delle basse vie aeree, indipendentemente dalla loro tipologia] *

Posto.il.cut.off.di.età.considerato.adeguato..in.quali.casi.effettui.la.diagnosi.di.bronchiolite.[T.Prodromi di infezione delle alte vie aeree e successivi segni di distress respiratorio con rumori umidi diffusi all’auscultazione delle basse vie aeree (per esempio rantoli o crepitii)]

---

Signif. codes: 0 '***' 0.001 '**' 0.01 '*' 0.05 '.' 0.1 ' ' 1

(Dispersion parameter for binomial family taken to be 1)

Null deviance: 423.88 on 305 degrees of freedom

Residual deviance: 407.04 on 299 degrees of freedom

AIC: 421.04

Number of Fisher Scoring iterations: 4

> exp(coef(GLM.2)) # Exponentiated coefficients ("odds ratios")

(Intercept)

0.8726954

Qual.è.il.limite.superiore.di.età.che.consideri.per.diagnosticare.la.bronchiolite..[T.≤24 mesi]

1.0722159

Qual.è.il.limite.superiore.di.età.che.consideri.per.diagnosticare.la.bronchiolite..[T.≤36 mesi]

0.1266411

Qual.è.il.limite.superiore.di.età.che.consideri.per.diagnosticare.la.bronchiolite..[T.≤6 mesi]

0.7109354

Qual.è.il.limite.superiore.di.età.che.consideri.per.diagnosticare.la.bronchiolite..[T.Non considero nessun limite superiore di età]

0.6469673

Posto.il.cut.off.di.età.considerato.adeguato..in.quali.casi.effettui.la.diagnosi.di.bronchiolite.[T.Prodromi di infezione delle alte vie aeree e successivi rumori diffusi all’auscultazione delle basse vie aeree, indipendentemente dalla loro tipologia]

2.4192055

Posto.il.cut.off.di.età.considerato.adeguato..in.quali.casi.effettui.la.diagnosi.di.bronchiolite.[T.Prodromi di infezione delle alte vie aeree e successivi segni di distress respiratorio con rumori umidi diffusi all’auscultazione delle basse vie aeree (per esempio rantoli o crepitii)]

1.4336610

> Confint(GLM.2, level=0.95, type="LR")

Estimate

(Intercept) -0.13616870

Qual.è.il.limite.superiore.di.età.che.consideri.per.diagnosticare.la.bronchiolite..[T.≤24 mesi] 0.06972745

Qual.è.il.limite.superiore.di.età.che.consideri.per.diagnosticare.la.bronchiolite..[T.≤36 mesi] -2.06639781

Qual.è.il.limite.superiore.di.età.che.consideri.per.diagnosticare.la.bronchiolite..[T.≤6 mesi] -0.34117377

Qual.è.il.limite.superiore.di.età.che.consideri.per.diagnosticare.la.bronchiolite..[T.Non considero nessun limite superiore di età] -0.43545954

Posto.il.cut.off.di.età.considerato.adeguato..in.quali.casi.effettui.la.diagnosi.di.bronchiolite.[T.Prodromi di infezione delle alte vie aeree e successivi rumori diffusi all’auscultazione delle basse vie aeree, indipendentemente dalla loro tipologia] 0.88343920

Posto.il.cut.off.di.età.considerato.adeguato..in.quali.casi.effettui.la.diagnosi.di.bronchiolite.[T.Prodromi di infezione delle alte vie aeree e successivi segni di distress respiratorio con rumori umidi diffusi all’auscultazione delle basse vie aeree (per esempio rantoli o crepitii)] 0.36023130

2.5 %

(Intercept) -0.5648782

Qual.è.il.limite.superiore.di.età.che.consideri.per.diagnosticare.la.bronchiolite..[T.≤24 mesi] -0.4254117

Qual.è.il.limite.superiore.di.età.che.consideri.per.diagnosticare.la.bronchiolite..[T.≤36 mesi] -3.9796140

Qual.è.il.limite.superiore.di.età.che.consideri.per.diagnosticare.la.bronchiolite..[T.≤6 mesi] -1.4345810

Qual.è.il.limite.superiore.di.età.che.consideri.per.diagnosticare.la.bronchiolite..[T.Non considero nessun limite superiore di età] -1.5132822

Posto.il.cut.off.di.età.considerato.adeguato..in.quali.casi.effettui.la.diagnosi.di.bronchiolite.[T.Prodromi di infezione delle alte vie aeree e successivi rumori diffusi all’auscultazione delle basse vie aeree, indipendentemente dalla loro tipologia] 0.2217070

Posto.il.cut.off.di.età.considerato.adeguato..in.quali.casi.effettui.la.diagnosi.di.bronchiolite.[T.Prodromi di infezione delle alte vie aeree e successivi segni di distress respiratorio con rumori umidi diffusi all’auscultazione delle basse vie aeree (per esempio rantoli o crepitii)] -0.1452676

97.5 %

(Intercept) 0.2896774

Qual.è.il.limite.superiore.di.età.che.consideri.per.diagnosticare.la.bronchiolite..[T.≤24 mesi] 0.5652777

Qual.è.il.limite.superiore.di.età.che.consideri.per.diagnosticare.la.bronchiolite..[T.≤36 mesi] -0.6706935

Qual.è.il.limite.superiore.di.età.che.consideri.per.diagnosticare.la.bronchiolite..[T.≤6 mesi] 0.7152990

Qual.è.il.limite.superiore.di.età.che.consideri.per.diagnosticare.la.bronchiolite..[T.Non considero nessun limite superiore di età] 0.5931401

Posto.il.cut.off.di.età.considerato.adeguato..in.quali.casi.effettui.la.diagnosi.di.bronchiolite.[T.Prodromi di infezione delle alte vie aeree e successivi rumori diffusi all’auscultazione delle basse vie aeree, indipendentemente dalla loro tipologia] 1.5751168

Posto.il.cut.off.di.età.considerato.adeguato..in.quali.casi.effettui.la.diagnosi.di.bronchiolite.[T.Prodromi di infezione delle alte vie aeree e successivi segni di distress respiratorio con rumori umidi diffusi all’auscultazione delle basse vie aeree (per esempio rantoli o crepitii)] 0.8693986

> Confint(GLM.2, level=0.95, type="LR", exponentiate=TRUE)

Exponentiated Coefficients and Confidence Bounds

Estimate

(Intercept) 0.8726954

Qual.è.il.limite.superiore.di.età.che.consideri.per.diagnosticare.la.bronchiolite..[T.≤24 mesi] 1.0722159

Qual.è.il.limite.superiore.di.età.che.consideri.per.diagnosticare.la.bronchiolite..[T.≤36 mesi] 0.1266411

Qual.è.il.limite.superiore.di.età.che.consideri.per.diagnosticare.la.bronchiolite..[T.≤6 mesi] 0.7109354

Qual.è.il.limite.superiore.di.età.che.consideri.per.diagnosticare.la.bronchiolite..[T.Non considero nessun limite superiore di età] 0.6469673

Posto.il.cut.off.di.età.considerato.adeguato..in.quali.casi.effettui.la.diagnosi.di.bronchiolite.[T.Prodromi di infezione delle alte vie aeree e successivi rumori diffusi all’auscultazione delle basse vie aeree, indipendentemente dalla loro tipologia] 2.4192055

Posto.il.cut.off.di.età.considerato.adeguato..in.quali.casi.effettui.la.diagnosi.di.bronchiolite.[T.Prodromi di infezione delle alte vie aeree e successivi segni di distress respiratorio con rumori umidi diffusi all’auscultazione delle basse vie aeree (per esempio rantoli o crepitii)] 1.4336610

2.5 %

(Intercept) 0.56842938

Qual.è.il.limite.superiore.di.età.che.consideri.per.diagnosticare.la.bronchiolite..[T.≤24 mesi] 0.65350067

Qual.è.il.limite.superiore.di.età.che.consideri.per.diagnosticare.la.bronchiolite..[T.≤36 mesi] 0.01869285

Qual.è.il.limite.superiore.di.età.che.consideri.per.diagnosticare.la.bronchiolite..[T.≤6 mesi] 0.23821516

Qual.è.il.limite.superiore.di.età.che.consideri.per.diagnosticare.la.bronchiolite..[T.Non considero nessun limite superiore di età] 0.22018609

Posto.il.cut.off.di.età.considerato.adeguato..in.quali.casi.effettui.la.diagnosi.di.bronchiolite.[T.Prodromi di infezione delle alte vie aeree e successivi rumori diffusi all’auscultazione delle basse vie aeree, indipendentemente dalla loro tipologia] 1.24820557

Posto.il.cut.off.di.età.considerato.adeguato..in.quali.casi.effettui.la.diagnosi.di.bronchiolite.[T.Prodromi di infezione delle alte vie aeree e successivi segni di distress respiratorio con rumori umidi diffusi all’auscultazione delle basse vie aeree (per esempio rantoli o crepitii)] 0.86479087

97.5 %

(Intercept) 1.3359964

Qual.è.il.limite.superiore.di.età.che.consideri.per.diagnosticare.la.bronchiolite..[T.≤24 mesi] 1.7599365

Qual.è.il.limite.superiore.di.età.che.consideri.per.diagnosticare.la.bronchiolite..[T.≤36 mesi] 0.5113539

Qual.è.il.limite.superiore.di.età.che.consideri.per.diagnosticare.la.bronchiolite..[T.≤6 mesi] 2.0447981

Qual.è.il.limite.superiore.di.età.che.consideri.per.diagnosticare.la.bronchiolite..[T.Non considero nessun limite superiore di età] 1.8096619

Posto.il.cut.off.di.età.considerato.adeguato..in.quali.casi.effettui.la.diagnosi.di.bronchiolite.[T.Prodromi di infezione delle alte vie aeree e successivi rumori diffusi all’auscultazione delle basse vie aeree, indipendentemente dalla loro tipologia] 4.8313059

Posto.il.cut.off.di.età.considerato.adeguato..in.quali.casi.effettui.la.diagnosi.di.bronchiolite.[T.Prodromi di infezione delle alte vie aeree e successivi segni di distress respiratorio con rumori umidi diffusi all’auscultazione delle basse vie aeree (per esempio rantoli o crepitii)] 2.3854758

**RLOGS_STEROIDS (age and symptoms)**

> summary(GLM.3)

Call:

glm(formula = Nella.gestione.ambulatoriale.della.bronchiolite.quali.indicazioni.routinariamente.fornisci.ai.genitori...Uso.di.steroidi.inalatori.o.per.bocca. ~

Qual.è.il.limite.superiore.di.età.che.consideri.per.diagnosticare.la.bronchiolite.. +

Posto.il.cut.off.di.età.considerato.adeguato..in.quali.casi.effettui.la.diagnosi.di.bronchiolite.,

family = binomial(logit), data = BR)

Coefficients:

Estimate

(Intercept) -0.48275

Qual.è.il.limite.superiore.di.età.che.consideri.per.diagnosticare.la.bronchiolite..[T.≤24 mesi] -0.01765

Qual.è.il.limite.superiore.di.età.che.consideri.per.diagnosticare.la.bronchiolite..[T.≤36 mesi] 0.91982

Qual.è.il.limite.superiore.di.età.che.consideri.per.diagnosticare.la.bronchiolite..[T.≤6 mesi] -0.03823

Qual.è.il.limite.superiore.di.età.che.consideri.per.diagnosticare.la.bronchiolite..[T.Non considero nessun limite superiore di età] 1.12855

Posto.il.cut.off.di.età.considerato.adeguato..in.quali.casi.effettui.la.diagnosi.di.bronchiolite.[T.Prodromi di infezione delle alte vie aeree e successivi rumori diffusi all’auscultazione delle basse vie aeree, indipendentemente dalla loro tipologia] -1.17157

Posto.il.cut.off.di.età.considerato.adeguato..in.quali.casi.effettui.la.diagnosi.di.bronchiolite.[T.Prodromi di infezione delle alte vie aeree e successivi segni di distress respiratorio con rumori umidi diffusi all’auscultazione delle basse vie aeree (per esempio rantoli o crepitii)] -0.48837

Std. Error

(Intercept) 0.22709

Qual.è.il.limite.superiore.di.età.che.consideri.per.diagnosticare.la.bronchiolite..[T.≤24 mesi] 0.27524

Qual.è.il.limite.superiore.di.età.che.consideri.per.diagnosticare.la.bronchiolite..[T.≤36 mesi] 0.61236

Qual.è.il.limite.superiore.di.età.che.consideri.per.diagnosticare.la.bronchiolite..[T.≤6 mesi] 0.58027

Qual.è.il.limite.superiore.di.età.che.consideri.per.diagnosticare.la.bronchiolite..[T.Non considero nessun limite superiore di età] 0.53804

Posto.il.cut.off.di.età.considerato.adeguato..in.quali.casi.effettui.la.diagnosi.di.bronchiolite.[T.Prodromi di infezione delle alte vie aeree e successivi rumori diffusi all’auscultazione delle basse vie aeree, indipendentemente dalla loro tipologia] 0.40083

Posto.il.cut.off.di.età.considerato.adeguato..in.quali.casi.effettui.la.diagnosi.di.bronchiolite.[T.Prodromi di infezione delle alte vie aeree e successivi segni di distress respiratorio con rumori umidi diffusi all’auscultazione delle basse vie aeree (per esempio rantoli o crepitii)] 0.27368

z value

(Intercept) -2.126

Qual.è.il.limite.superiore.di.età.che.consideri.per.diagnosticare.la.bronchiolite..[T.≤24 mesi] -0.064

Qual.è.il.limite.superiore.di.età.che.consideri.per.diagnosticare.la.bronchiolite..[T.≤36 mesi] 1.502

Qual.è.il.limite.superiore.di.età.che.consideri.per.diagnosticare.la.bronchiolite..[T.≤6 mesi] -0.066

Qual.è.il.limite.superiore.di.età.che.consideri.per.diagnosticare.la.bronchiolite..[T.Non considero nessun limite superiore di età] 2.098

Posto.il.cut.off.di.età.considerato.adeguato..in.quali.casi.effettui.la.diagnosi.di.bronchiolite.[T.Prodromi di infezione delle alte vie aeree e successivi rumori diffusi all’auscultazione delle basse vie aeree, indipendentemente dalla loro tipologia] -2.923

Posto.il.cut.off.di.età.considerato.adeguato..in.quali.casi.effettui.la.diagnosi.di.bronchiolite.[T.Prodromi di infezione delle alte vie aeree e successivi segni di distress respiratorio con rumori umidi diffusi all’auscultazione delle basse vie aeree (per esempio rantoli o crepitii)] -1.784

Pr(>|z|)

(Intercept) 0.03352

Qual.è.il.limite.superiore.di.età.che.consideri.per.diagnosticare.la.bronchiolite..[T.≤24 mesi] 0.94886

Qual.è.il.limite.superiore.di.età.che.consideri.per.diagnosticare.la.bronchiolite..[T.≤36 mesi] 0.13307

Qual.è.il.limite.superiore.di.età.che.consideri.per.diagnosticare.la.bronchiolite..[T.≤6 mesi] 0.94747

Qual.è.il.limite.superiore.di.età.che.consideri.per.diagnosticare.la.bronchiolite..[T.Non considero nessun limite superiore di età] 0.03595

Posto.il.cut.off.di.età.considerato.adeguato..in.quali.casi.effettui.la.diagnosi.di.bronchiolite.[T.Prodromi di infezione delle alte vie aeree e successivi rumori diffusi all’auscultazione delle basse vie aeree, indipendentemente dalla loro tipologia] 0.00347

Posto.il.cut.off.di.età.considerato.adeguato..in.quali.casi.effettui.la.diagnosi.di.bronchiolite.[T.Prodromi di infezione delle alte vie aeree e successivi segni di distress respiratorio con rumori umidi diffusi all’auscultazione delle basse vie aeree (per esempio rantoli o crepitii)] 0.07435

(Intercept) *

Qual.è.il.limite.superiore.di.età.che.consideri.per.diagnosticare.la.bronchiolite..[T.≤24 mesi]

Qual.è.il.limite.superiore.di.età.che.consideri.per.diagnosticare.la.bronchiolite..[T.≤36 mesi]

Qual.è.il.limite.superiore.di.età.che.consideri.per.diagnosticare.la.bronchiolite..[T.≤6 mesi]

Qual.è.il.limite.superiore.di.età.che.consideri.per.diagnosticare.la.bronchiolite..[T.Non considero nessun limite superiore di età] *

Posto.il.cut.off.di.età.considerato.adeguato..in.quali.casi.effettui.la.diagnosi.di.bronchiolite.[T.Prodromi di infezione delle alte vie aeree e successivi rumori diffusi all’auscultazione delle basse vie aeree, indipendentemente dalla loro tipologia] **

Posto.il.cut.off.di.età.considerato.adeguato..in.quali.casi.effettui.la.diagnosi.di.bronchiolite.[T.Prodromi di infezione delle alte vie aeree e successivi segni di distress respiratorio con rumori umidi diffusi all’auscultazione delle basse vie aeree (per esempio rantoli o crepitii)] .

---

Signif. codes: 0 '***' 0.001 '**' 0.01 '*' 0.05 '.' 0.1 ' ' 1

(Dispersion parameter for binomial family taken to be 1)

Null deviance: 385.25 on 305 degrees of freedom

Residual deviance: 368.11 on 299 degrees of freedom

AIC: 382.11

Number of Fisher Scoring iterations: 4

> exp(coef(GLM.3)) # Exponentiated coefficients ("odds ratios")

(Intercept)

0.6170811

Qual.è.il.limite.superiore.di.età.che.consideri.per.diagnosticare.la.bronchiolite..[T.≤24 mesi]

0.9825000

Qual.è.il.limite.superiore.di.età.che.consideri.per.diagnosticare.la.bronchiolite..[T.≤36 mesi]

2.5088495

Qual.è.il.limite.superiore.di.età.che.consideri.per.diagnosticare.la.bronchiolite..[T.≤6 mesi]

0.9624879

Qual.è.il.limite.superiore.di.età.che.consideri.per.diagnosticare.la.bronchiolite..[T.Non considero nessun limite superiore di età]

3.0911629

Posto.il.cut.off.di.età.considerato.adeguato..in.quali.casi.effettui.la.diagnosi.di.bronchiolite.[T.Prodromi di infezione delle alte vie aeree e successivi rumori diffusi all’auscultazione delle basse vie aeree, indipendentemente dalla loro tipologia]

0.3098814

Posto.il.cut.off.di.età.considerato.adeguato..in.quali.casi.effettui.la.diagnosi.di.bronchiolite.[T.Prodromi di infezione delle alte vie aeree e successivi segni di distress respiratorio con rumori umidi diffusi all’auscultazione delle basse vie aeree (per esempio rantoli o crepitii)]

0.6136276

> Confint(GLM.3, level=0.95, type="LR")

Estimate

(Intercept) -0.48275482

Qual.è.il.limite.superiore.di.età.che.consideri.per.diagnosticare.la.bronchiolite..[T.≤24 mesi] -0.01765489

Qual.è.il.limite.superiore.di.età.che.consideri.per.diagnosticare.la.bronchiolite..[T.≤36 mesi] 0.91982429

Qual.è.il.limite.superiore.di.età.che.consideri.per.diagnosticare.la.bronchiolite..[T.≤6 mesi] -0.03823382

Qual.è.il.limite.superiore.di.età.che.consideri.per.diagnosticare.la.bronchiolite..[T.Non considero nessun limite superiore di età] 1.12854737

Posto.il.cut.off.di.età.considerato.adeguato..in.quali.casi.effettui.la.diagnosi.di.bronchiolite.[T.Prodromi di infezione delle alte vie aeree e successivi rumori diffusi all’auscultazione delle basse vie aeree, indipendentemente dalla loro tipologia] -1.17156559

Posto.il.cut.off.di.età.considerato.adeguato..in.quali.casi.effettui.la.diagnosi.di.bronchiolite.[T.Prodromi di infezione delle alte vie aeree e successivi segni di distress respiratorio con rumori umidi diffusi all’auscultazione delle basse vie aeree (per esempio rantoli o crepitii)] -0.48836712

2.5 %

(Intercept) -0.93553105

Qual.è.il.limite.superiore.di.età.che.consideri.per.diagnosticare.la.bronchiolite..[T.≤24 mesi] -0.55855901

Qual.è.il.limite.superiore.di.età.che.consideri.per.diagnosticare.la.bronchiolite..[T.≤36 mesi] -0.30819151

Qual.è.il.limite.superiore.di.età.che.consideri.per.diagnosticare.la.bronchiolite..[T.≤6 mesi] -1.25988851

Qual.è.il.limite.superiore.di.età.che.consideri.per.diagnosticare.la.bronchiolite..[T.Non considero nessun limite superiore di età] 0.08450368

Posto.il.cut.off.di.età.considerato.adeguato..in.quali.casi.effettui.la.diagnosi.di.bronchiolite.[T.Prodromi di infezione delle alte vie aeree e successivi rumori diffusi all’auscultazione delle basse vie aeree, indipendentemente dalla loro tipologia] -2.00507036

Posto.il.cut.off.di.età.considerato.adeguato..in.quali.casi.effettui.la.diagnosi.di.bronchiolite.[T.Prodromi di infezione delle alte vie aeree e successivi segni di distress respiratorio con rumori umidi diffusi all’auscultazione delle basse vie aeree (per esempio rantoli o crepitii)] -1.03102007

97.5 %

(Intercept) -0.04251265

Qual.è.il.limite.superiore.di.età.che.consideri.per.diagnosticare.la.bronchiolite..[T.≤24 mesi] 0.52275338

Qual.è.il.limite.superiore.di.età.che.consideri.per.diagnosticare.la.bronchiolite..[T.≤36 mesi] 2.13736893

Qual.è.il.limite.superiore.di.età.che.consideri.per.diagnosticare.la.bronchiolite..[T.≤6 mesi] 1.05977808

Qual.è.il.limite.superiore.di.età.che.consideri.per.diagnosticare.la.bronchiolite..[T.Non considero nessun limite superiore di età] 2.22410702

Posto.il.cut.off.di.età.considerato.adeguato..in.quali.casi.effettui.la.diagnosi.di.bronchiolite.[T.Prodromi di infezione delle alte vie aeree e successivi rumori diffusi all’auscultazione delle basse vie aeree, indipendentemente dalla loro tipologia] -0.42042741

Posto.il.cut.off.di.età.considerato.adeguato..in.quali.casi.effettui.la.diagnosi.di.bronchiolite.[T.Prodromi di infezione delle alte vie aeree e successivi segni di distress respiratorio con rumori umidi diffusi all’auscultazione delle basse vie aeree (per esempio rantoli o crepitii)] 0.04410948

> Confint(GLM.3, level=0.95, type="LR", exponentiate=TRUE)

Exponentiated Coefficients and Confidence Bounds

Estimate

(Intercept) 0.6170811

Qual.è.il.limite.superiore.di.età.che.consideri.per.diagnosticare.la.bronchiolite..[T.≤24 mesi] 0.9825000

Qual.è.il.limite.superiore.di.età.che.consideri.per.diagnosticare.la.bronchiolite..[T.≤36 mesi] 2.5088495

Qual.è.il.limite.superiore.di.età.che.consideri.per.diagnosticare.la.bronchiolite..[T.≤6 mesi] 0.9624879

Qual.è.il.limite.superiore.di.età.che.consideri.per.diagnosticare.la.bronchiolite..[T.Non considero nessun limite superiore di età] 3.0911629

Posto.il.cut.off.di.età.considerato.adeguato..in.quali.casi.effettui.la.diagnosi.di.bronchiolite.[T.Prodromi di infezione delle alte vie aeree e successivi rumori diffusi all’auscultazione delle basse vie aeree, indipendentemente dalla loro tipologia] 0.3098814

Posto.il.cut.off.di.età.considerato.adeguato..in.quali.casi.effettui.la.diagnosi.di.bronchiolite.[T.Prodromi di infezione delle alte vie aeree e successivi segni di distress respiratorio con rumori umidi diffusi all’auscultazione delle basse vie aeree (per esempio rantoli o crepitii)] 0.6136276

2.5 %

(Intercept) 0.3923774

Qual.è.il.limite.superiore.di.età.che.consideri.per.diagnosticare.la.bronchiolite..[T.≤24 mesi] 0.5720328

Qual.è.il.limite.superiore.di.età.che.consideri.per.diagnosticare.la.bronchiolite..[T.≤36 mesi] 0.7347746

Qual.è.il.limite.superiore.di.età.che.consideri.per.diagnosticare.la.bronchiolite..[T.≤6 mesi] 0.2836857

Qual.è.il.limite.superiore.di.età.che.consideri.per.diagnosticare.la.bronchiolite..[T.Non considero nessun limite superiore di età] 1.0881769

Posto.il.cut.off.di.età.considerato.adeguato..in.quali.casi.effettui.la.diagnosi.di.bronchiolite.[T.Prodromi di infezione delle alte vie aeree e successivi rumori diffusi all’auscultazione delle basse vie aeree, indipendentemente dalla loro tipologia] 0.1346508

Posto.il.cut.off.di.età.considerato.adeguato..in.quali.casi.effettui.la.diagnosi.di.bronchiolite.[T.Prodromi di infezione delle alte vie aeree e successivi segni di distress respiratorio con rumori umidi diffusi all’auscultazione delle basse vie aeree (per esempio rantoli o crepitii)] 0.3566430

97.5 %

(Intercept) 0.9583783

Qual.è.il.limite.superiore.di.età.che.consideri.per.diagnosticare.la.bronchiolite..[T.≤24 mesi] 1.6866653

Qual.è.il.limite.superiore.di.età.che.consideri.per.diagnosticare.la.bronchiolite..[T.≤36 mesi] 8.4771044

Qual.è.il.limite.superiore.di.età.che.consideri.per.diagnosticare.la.bronchiolite..[T.≤6 mesi] 2.8857305

Qual.è.il.limite.superiore.di.età.che.consideri.per.diagnosticare.la.bronchiolite..[T.Non considero nessun limite superiore di età] 9.2452233

Posto.il.cut.off.di.età.considerato.adeguato..in.quali.casi.effettui.la.diagnosi.di.bronchiolite.[T.Prodromi di infezione delle alte vie aeree e successivi rumori diffusi all’auscultazione delle basse vie aeree, indipendentemente dalla loro tipologia] 0.6567661

Posto.il.cut.off.di.età.considerato.adeguato..in.quali.casi.effettui.la.diagnosi.di.bronchiolite.[T.Prodromi di infezione delle alte vie aeree e successivi segni di distress respiratorio con rumori umidi diffusi all’auscultazione delle basse vie aere
