# Supplementary material for: Diagnosis, management, and prevention of bronchiolitis in primary care: a survey of Italian family paediatricians
Source: Ital J Pediatr. 2025 Nov 19;51:305. doi: 10.1186/s13052-025-02152-y (PMC12628587; doi:10.1186/s13052-025-02152-y)
Supplement: Supplementary file 3 — Supplementary Material 3 [file 13052_2025_2152_MOESM3_ESM.doc]

**Diagnosis, Management, and Prevention of Bronchiolitis in Primary Care: A Survey of Italian Family Paediatricians**

**STROBE CHECKLIST**

STROBE Statement—Checklist of items that should be included in reports of ***cross-sectional studies***

|  | Item No | Recommendation |
| --- | --- | --- |
| **Title and abstract** | 1 | (*a*) Indicate the study’s design with a commonly used term in the title or the abstract  **Page 1** |
|  |  | (*b*) Provide in the abstract an informative and balanced summary of what was done and what was found  **Page 2** |
| Introduction | | |
| Background/rationale | 2 | Explain the scientific background and rationale for the investigation being reported  **Page 3** |
| Objectives | 3 | State specific objectives, including any prespecified hypotheses  **Page 3** |
| Methods | | |
| Study design | 4 | Present key elements of study design early in the paper  **Page 4** |
| Setting | 5 | Describe the setting, locations, and relevant dates, including periods of recruitment, exposure, follow-up, and data collection  **Page 4** |
| Participants | 6 | (*a*) Give the eligibility criteria, and the sources and methods of selection of participants  **Page 4** |
| Variables | 7 | Clearly define all outcomes, exposures, predictors, potential confounders, and effect modifiers. Give diagnostic criteria, if applicable  **Page 4** |
| Data sources/ measurement | 8* | For each variable of interest, give sources of data and details of methods of assessment (measurement). Describe comparability of assessment methods if there is more than one group |
| Bias | 9 | Describe any efforts to address potential sources of bias  **Page 4-5** |
| Study size | 10 | Explain how the study size was arrived at  **Page 5** |
| Quantitative variables | 11 | Explain how quantitative variables were handled in the analyses. If applicable, describe which groupings were chosen and why  **Not applicable** |
| Statistical methods | 12 | (*a*) Describe all statistical methods, including those used to control for confounding **Page 4-5** |
|  |  | (*b*) Describe any methods used to examine subgroups and interactions **Page 4-5** |
|  |  | (*c*) Explain how missing data were addressed **Not applicable** |
|  |  | (*d*) If applicable, describe analytical methods taking account of sampling strategy **Not applicable** |
|  |  | (*e*) Describe any sensitivity analyses **Not applicable** |
| Results | | |
| Participants | 13* | (a) Report numbers of individuals at each stage of study—eg numbers potentially eligible, examined for eligibility, confirmed eligible, included in the study, completing follow-up, and analysed **Page 5** |
|  |  | (b) Give reasons for non-participation at each stage **Page 5** |
|  |  | (c) Consider use of a flow diagram |
| Descriptive data | 14* | (a) Give characteristics of study participants (eg demographic, clinical, social) and information on exposures and potential confounders **Not applicable** |
|  |  | (b) Indicate number of participants with missing data for each variable of interest **Tables** |
| Outcome data | 15* | Report numbers of outcome events or summary measures **Page 5-6** |
| Main results | 16 | (*a*) Give unadjusted estimates and, if applicable, confounder-adjusted estimates and their precision (eg, 95% confidence interval). Make clear which confounders were adjusted for and why they were included **Page 5-6-7** |
|  |  | (*b*) Report category boundaries when continuous variables were categorized **Not applicable** |
|  |  | (*c*) If relevant, consider translating estimates of relative risk into absolute risk for a meaningful time period **Not applicable** |
| Other analyses | 17 | Report other analyses done—eg analyses of subgroups and interactions, and sensitivity analyses **Page 6-7** |
| Discussion | | |
| Key results | 18 | Summarise key results with reference to study objectives **Page 7** |
| Limitations | 19 | Discuss limitations of the study, taking into account sources of potential bias or imprecision. Discuss both direction and magnitude of any potential bias **Page 8** |
| Interpretation | 20 | Give a cautious overall interpretation of results considering objectives, limitations, multiplicity of analyses, results from similar studies, and other relevant evidence **Page 8-9** |
| Generalisability | 21 | Discuss the generalisability (external validity) of the study results **Page 9** |
| Other information | | |
| Funding | 22 | Give the source of funding and the role of the funders for the present study and, if applicable, for the original study on which the present article is based **Page 10** |

*Give information separately for exposed and unexposed groups.
